# Neuropharmacological Effects of Quercetin: A Literature-Based Review

**DOI:** 10.3389/fphar.2021.665031

**Published:** 2021-06-17

**Authors:** Md. Shahazul Islam, Cristina Quispe, Rajib Hossain, Muhammad Torequl Islam, Ahmed Al-Harrasi, Ahmed Al-Rawahi, Miquel Martorell, Assem Mamurova, Ainur Seilkhan, Nazgul Altybaeva, Bagila Abdullayeva, Anca Oana Docea, Daniela Calina, Javad Sharifi-Rad

**Affiliations:** ^1^ Department of Pharmacy, Life Science Faculty, Bangabandhu Sheikh Mujibur Rahman Science and Technology University, Gopalganj, Bangladesh; ^2^ Facultad de Ciencias de La Salud, Universidad Arturo Prat, Iquique, Chile; ^3^ Natural and Medical Sciences Research Centre, University of Nizwa, Nizwa, Oman; ^4^ Department of Nutrition and Dietetics, Faculty of Pharmacy, Centre for Healthy Living, University of Concepción, Concepción, Chile; ^5^ Department of Biodiversity of Bioresources, Al-Farabi Kazakh National University, Almaty, Kazakhstan; ^6^ Educational program, Geography, Environment and Service sector, Abai Kazakh National Pedagogical University, Kazakhstan, Almaty, Kazakhstan; ^7^ Biomedical Research Centre, Al-Farabi Kazakh National University, Almaty, Kazakhstan; ^8^ Department of Molecular Biology and Genetics, Al-Farabi Kazakh National University, Almaty, Kazakhstan; ^9^ Department of Toxicology, University of Medicine and Pharmacy of Craiova, Craiova, Romania; ^10^ Department of Clinical Pharmacy, University of Medicine and Pharmacy of Craiova, Craiova, Romania; ^11^ Phytochemistry Research Center, Shahid Beheshti University of Medical Sciences, Tehran, Iran

**Keywords:** quercetin, neuropharmacological effects, neural damage, signaling pathways, mechanisms, neurodegenerative disorders

## Abstract

Quercetin (QUR) is a natural bioactive flavonoid that has been lately very studied for its beneficial properties in many pathologies. Its neuroprotective effects have been demonstrated in many *in vitro* studies, as well as *in vivo* animal experiments and human trials. QUR protects the organism against neurotoxic chemicals and also can prevent the evolution and development of neuronal injury and neurodegeneration. The present work aimed to summarize the literature about the neuroprotective effect of QUR using known database sources. Besides, this review focuses on the assessment of the potential utilization of QUR as a complementary or alternative medicine for preventing and treating neurodegenerative diseases. An up-to-date search was conducted in PubMed, Science Direct and Google Scholar for published work dealing with the neuroprotective effects of QUR against neurotoxic chemicals or in neuronal injury, and in the treatment of neurodegenerative diseases. Findings suggest that QUR possess neuropharmacological protective effects in neurodegenerative brain disorders such as Alzheimer’s disease, Amyloid β peptide, Parkinson’s disease, Huntington's disease, multiple sclerosis, and amyotrophic lateral sclerosis. In summary, this review emphasizes the neuroprotective effects of QUR and its advantages in being used in complementary medicine for the prevention and treatment o of different neurodegenerative diseases.

## Introduction

Neuropharmacology is the investigation of what medications mean for cell work in the sensory system and the neural components with its mechanisms through which they impact behavior (Yeung et al., 2018). There is plethora of neurological conditions such as neuropathic pain, neurodegenerative diseases such as Parkinson's disease (PD) and Alzheimer's disease (AD), psychological disorders.

**FIGURE 1:**
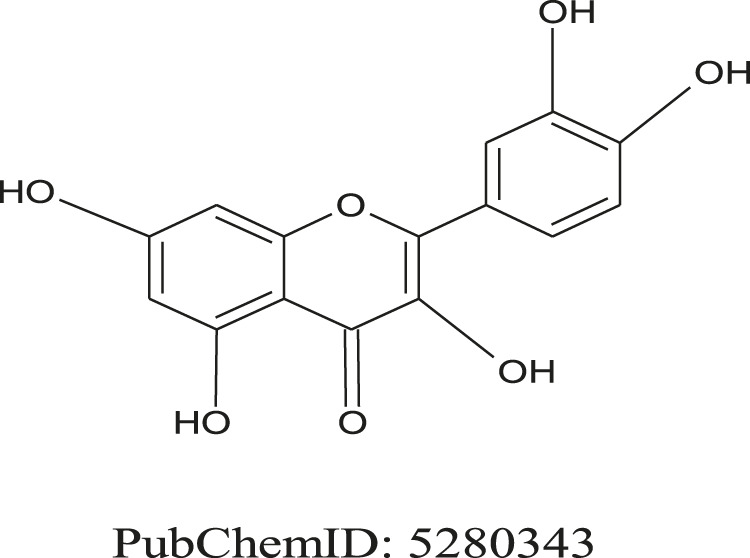
Chemical structure of 2-(3,4-dihydroxy phenyl)-3,5,7-trihydroxychromen-4-one (Quercetin).

The use of medicinal plants for the prevention or treatment of neurological diseases has a long history (Salehi et al., 2021a). Due to their active compounds such as flavonoids, curcumin, lycopene, resveratrol, sesamol, etc. several natural products have been tested and showed their neuroprotective effects *in vitro* and *in vivo* studies (Hanganu et al., 2019; Burlec et al., 2020). Quercetin (QUR, C_15_H_10_O_7_), also known as 3,3′,4′,5,7-pentahydroxyflavone (Figure 1), is a unique bioflavonoid, plentifully found in various leafy foods and fruits e.g. apples, berries, chokeberries cilantro, dill, escapades, lingonberries, lovage, onions (Yang et al., 2020; Sharifi-Rad et al., 2021b). It exhibits several biopharmacological activities (Kawabata et al., 2015), including antioxidant (Chaudhary et al., 2015) and anti-inflammatory (Kim and Park, 2016; Zheng et al., 2016) activities, neuroprotective properties against CNS disorders, including memory impairment (Nassiri-Asl et al., 2013; Abdalla et al., 2014; Zhang et al., 2016), seizure (Nieoczym et al., 2014; Nassiri-Asl et al., 2016), Huntington's disease (HD) (Chakraborty et al., 2014), and PD (Gomez del Rio et al., 2013). QUR also exhibits anticonvulsant activity (Nassiri-Asl et al., 2013; Nassiri-Asl et al., 2016). QUR is clearly a polar auxin transport antagonist (Fischer et al., 1997).

This review focuses on emerging comprehension information to the preventive and therapeutic ability of this natural flavonoid against neurological and neurodegenerative diseases, along with its mechanisms of action. Additionally, we have also summarized the biological sources and other pharmacological activities of QUR.

## Review Methodology

This updated review covers the neuroprotective effects and potential alternative therapeutic options of QUR in neurological disorders. Scientific data on the neuroprotective effects of QUR were collected from online databases such as PubMed (https://www.ncbi.nlm.nih.gov/pubmed/), Science Direct (http://www.sciencedirect.com/), and Google Scholar (https://scholar.google.com/).

Search keywords were: “Quercetin”,“biodisponibility” “neuroprotective”, “neurodegenerative diseases”, “oxidative stress”, “neuroinflammation”, “tau protein”, “neurotrophic factors”, “amyloid beta”, “memory”, “learning”, “pharmaceutical formulations”, “QUR encapsulation”. The chemical structure was revised by consulting the open PubChem database (https://scholar.google.com/) and the scientific names of the plants were revised according to PlantList. For this updated review, were included: in extenso papers written in the English language, *in vitro* and *in vivo* experimental studies that showed the effective doses compared with control and studies hich highlighted the molecular mechanisms of the neuroprotective effects of QUR. Abstracts, communications, studies that included homoeopathic preparations or brain tumors associated with neurological/neurodegenerative diseases were excluded.

## Bioavailability of Quercetin

QUR is a flavonol known as a subcategory of flavonoids. These are plant pigments, known as phytonutrients with plenty of benefits for the body's health. This pigment can be found in many fruits, vegetables, plants and beverages such as berries, apples, cherries, red onions, tomatoes, broccoli and citrus, red wine and black (Suganthy et al., 2016). QUR can be assimilated by the daily consumption of fruits and vegetables (Khan et al., 2020). In vegetables, QUR is found in the highest amount in onions compared to other vegetables. One kilogram of onions contains around 300 mg of QUR (Khan et al., 2020; Kothari et al., 2020).

QUR has a bitter taste, low bioavailability due to poor solubility and absorption as well as rapid metabolism (Guo and Bruno, 2015; Khan et al., 2020). An *in vivo* study in pigs investigated the bioavailability and metabolism of QUR. An intravenous dose of 0.4 mg/kgc was initially administered and after seven days an oral dose of 50 mg/kgc. The results of this study showed that the apparent bioavailability of QUR was only 0.54 ± 0.19% when considering only free QUR, 8.6 ± 3.8% when measuring also the conjugated QUR in the intestinal wall and 17.0 ± 7.1% when considered also QUR metabolites (Ader et al., 2000).

QUR does not have the high efficiency of crossing the normal blood-brain barrier (Oliveira et al., 2021). Various methods have been tried to increase QUR’s bioavailability such as the enzymatic modification or nanoencapsulation (Pateiro et al., 2021).

Enzymatically modified isoquercitrin (EMIQ) has an increased bioavailability and is prepared using a natural enzymatic process that attaches polysaccharides to convert QUR, which has poor bioavailability, into a water-soluble form (Alpha-Glycosyl Isoquercitrin) (Omi et al., 2019). This method has the advantage of high absorption and superior bioavailability. According to pharmacokinetic data, the absorption of Isoquercetin is up to 40 times higher (Cmax) than that of QUR and reaches maximum levels in the bloodstream in just 15 min (Omi et al., 2019). Therefore, Isoquercitrine is much more bioactive than QUR and has a broader spectrum of therapeutic activity.

Nanotechnologies and target carriers are the solution to overcome the disadvantage of low solubility and bioavailability, increased metabolism and low brain penetration of QUR (Naseri et al., 2015). Modern research has shown that there are many possibilities for the penetration of bioactive compounds into the brain such as blood-mediated receptor-mediated transcytosis (BBB) and coupling with transferrin receptors found on the luminal side of capillary endothelial cells of the brain. Therefore, the inclusion of QUR in transferrin-functionalized liposomes is a solution to facilitate QUR penetration into the brain (Pinheiro et al., 2020).

New nanoencapsulation pharmaceutical formulations such as phytosomes have been tried for increasing QUR bioavailability. Phytosomes are obtained from phospholipids, which are from the same material that makes up cell membranes. By encapsulation in phytosomes, QUR can cross the cell membrane and is delivered directly inside the cell, making it more bioavailable and easy to be absorbed and used by the tissues. This technology is similar to liposomal entanglement technology (Riva et al., 2019). In a randomized clinical trial, researchers compared two doses of QUR phytosomes administered orally (providing 100 and 200 mg of QUR), along with a single 500 mg dose of QUR (Riva et al., 2019). The results showed that, when administered in phytosomes, this compound could be administered at one-fifth of the dose of traditional QUR and at the same time to obtain a10 times higher exposure. This study showed that when encapsulated in phytosmes, QUR has a bioavailability of 50 times higher compared to QUR standard products.

## Quercetin’s Effects in Neurological Diseases: Molecular Mechanisms and Signaling Pathways

### Quercetin Against Oxidative Stress

It is grounded by scientists that the human brain use 20% of the body’s daily intake of O_2_. The human brain has a significant amount of polyunsaturated fatty acids (Sharifi-Rad et al., 2021b). These along with transition metal ions and poor antioxidant enzymes makes the organ vulnerable in front of free radicals (Uttara et al., 2009; Buga et al., 2019; Aloizou et al., 2021). The neurotransmitters and excitatory amino acids act as a source of oxygen species like ROS, particularly present in the brain, promoting oxidative stress damage (Tsatsakis et al., 2019). ROS induce protein oxidation (Salehi et al., 2019a), lipid peroxidation (Salehi et al., 2019c), and neurons and glial cells leading to the death of neurons (Gilgun-Sherki et al., 2001). Moreover, aging established neuronal damage mediated by ROS cause neurodegeneration (Uttara et al., 2009; Alvarez-Arellano et al., 2020).

A series of studies have shown that the primary cause of neurodegenerative brain disease and vascular pathology is oxidative stress (Gandhi and Abramov, 2012; Sharifi-Rad et al., 2020d). Subsequently, the quest for a convincing instrument to counteract neuronal harm impacted by oxidative stress prompted the analysis of antioxidant molecules as a ROS scavenger (Feng and Wang, 2012; Salehi et al., 2020b).

**FIGURE 2:**
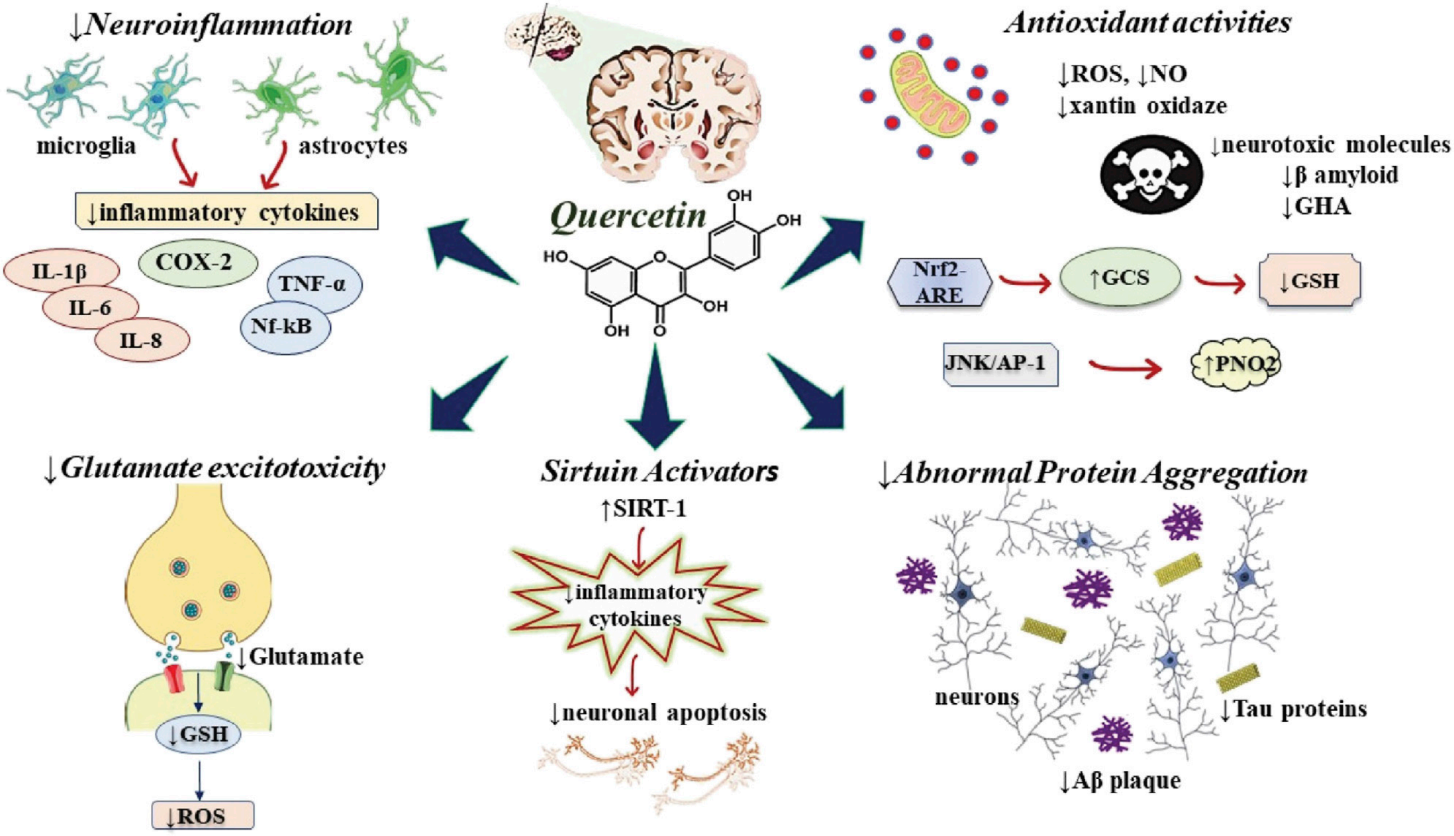
Diagram with possible neuroprotective mechanisms of QUR. The figure highlights the neuronal neuroprotective action in various signaling pathways that lead to the release of proinflammatory cytokines, neurodegeneration and cellular apoptosis. *Abbreviations:* ↑, increase; ↓, decrease; Aβ, amyloid beta-peptide; GCS, γ-glutamyl-cysteine synthetase; GSH, glutathione; IL, interleukin; JNK, c-Jun N-terminal kinase; NO, nitric oxide; Nrf2-ARE, Nuclear factor erythroid-derived 2-like 2- antioxidant responsive element; PNO2, paraoxygenase 2; ROS, reactive oxygen species; SIRT1, sirtuin 1; TNF-α, tumor necrosis factor-alfa.

Through scavenging oxygen radicals and metal chelating operations, QUR attenuates neuronal damage mediated by oxidative stress (Echeverry et al., 2010). The scavenging mechanism of QUR is followed by attenuating nitric oxide (NO) synthase and xanthine oxidase. Besides, QUR can activate the Nrf2-ARE signaling pathway and mitigate neuronal damage mediated by oxidative stress. γ-glutamyl-cysteine synthetase is elevated by Nrf2-ARE pathway for glutathione (GSH) synthesis (Arredondo et al., 2010) (Figure 2).

Several *in vitro* studies have shown that QUR, by its immediate and aberrant antioxidant action, enhances cell resistance to oxidative stress caused by hydrogen peroxide, β-amyloid peptide and 6-hydroxylamine (Ansari et al., 2009; Suematsu et al., 2011; Shokoohinia et al., 2015; Magalingam et al., 2016).

Omnipresently, a catalyst present in the human brain is paraoxonase 2 (PNO2), which protects the brain from neuroprotection by the reduction of injury caused by oxidation because they are located in mitochondria (Costa et al., 2014). QUR increases PNO2 expression in brain cells, macrophages, neurons, and striatal astrocytes, not only at the mRNA but also at the protein level (Boesch-Saadatmandi et al., 2009). The appropriate mechanism of expression of PNO2 by QUR can be associated with a high antioxidant effect or with the modification of JNK/AP-1 pathway. JNK/AP-1 pathway is known to enhance PNO2 expression (Granado-Serrano et al., 2010).

### Quercetin Against Neuroinflammatory Responses

For neurodegenerative diseases, inflammation plays a key role (Alam et al., 2016; Sharifi-Rad et al., 2020b). Neuroinflammation tends to be a secondary reaction triggered by early brain injury (trauma, cancer, amyloid beta-peptide (Aβ) and hyperphosphorylated tau) that cause significant neuronal damage (Glass et al., 2010; Sharifi-Rad et al., 2021a). Therefore, in the creation of different neuropathologies, inflammation is now considered as a driving factor (Smith et al., 2012; Tsoukalas et al., 2021). Neuron-like activation of microglia and astrocytes induces pro-inflammatory mediator expression including such as cytokines (interleukin (IL)-1β, tumor necrosis factor (TNF)-α, complementary components, acute-phase protein (Smith et al., 2012), which can stimulate inducible nitric oxide synthase (iNOS) and NO production with abnormal phagocytic activity (Lyman et al., 2014) (Figure 2). All these phages contribute to neuronal degeneration and neurodegenerative disease development such as AD (Heneka et al., 2015), PD (Rocha et al., 2015), HD (Hsiao et al., 2013), multiple sclerosis (MS) (Naegele and Martin, 2014; Padureanu et al., 2019), and amyotrophic lateral sclerosis (ALS) (Hooten et al., 2015). CNS demonstrates extreme prevalence to inflammatory stimuli by increased development of cytokines and ROS with increased phagocytic potential during aging (Hickman et al., 2013).

Neuroinflammation can be suppressed by anti-inflammatory agents that can stimulate NO development, cascades of glial activation and inflammatory cytokines and prevent neuronal death (Lee et al., 2008). Several *in vitro* and *in vivo* experiments have demonstrated that QUR has significant anti-inflammatory activities (Assi and El Sayed, 1998). In PC12 cells and zebrafish models, QUR blocked inflammation induced by the toxicity of 6-hydroxydopamine (6-OHDA) via repressing overproduction of NO, iNOS enzyme, and other inflammatory genes (Zhang et al., 2011).

Similarly, QUR inhibited lipopolysaccharide (LPS)/Interferon γ induced inflammation by abrogating iNOS expression (Chen et al., 2006), downregulating the extracellular signal-regulated kinase, c-Jun, N-terminal kinase, Akt, Src, Janus kinase-1, activating protein-1 (AP-1) (Kim et al., 2005), and enhancing the expression of heme oxygenase (HO)-1 (Sun et al., 2015). Besides, in astrocytes, QUR decreases pro-inflammatory cytokines (e.g., TNF-α and IL-1α) (Sharma et al., 2007) and diminished microglial activated neuronal cell death in microglial N9-neuronal (PC12) co-culture (Bureau et al., 2008).

By downregulating TLR4 and COX2, QUR along with β-cyclodextrin-dodecylcarbonate nanoparticles illustrated strong anti-inflammatory activity. These nanoparticles demonstrated improved blood-brain cognitive pathways and pharmacokinetics to target cells (Testa et al., 2014). In brain tissue, QUR attenuates microglial activation and inflammation-induced neuron death evoked by 1-methyl-4-phenylpyridinium (Sternberg et al., 2008), and IL-1β and monocyte chemoattractant protein (MCP)-1 expression (Jung et al., 2010).

Furthermore, astrocytes liberate pro-inflammatory cytokines (e.g., TNF-α, IL-1, IL-6) and neurotoxic factors and cause neuronal damage (Niranjan, 2014). QUR exerts a defensive effect on tert-butyl hydroperoxide and H2O2 toxicity in astrocytes via modulating ROS generation, decreasing apoptosis and boosting HO-1 transcription. Moreover, expression of glutamate-cysteine ligase (GCL) and GSH synthetase was increased dramatically by QUR, in astrocytes and attenuated inflammation by repression of IL-1β,6,8, monocyte chemoattractant protein-1 and ROS production release (Chen et al., 2006; Sharma et al., 2007). Therefore, QUR exhibits a potent anti neuroinflammatory activity, because QUR can repress TNF- α via amplifying the NFkB signaling pathway (Madhavan et al., 2009), with its antioxidant effect (Boesch-Saadatmandi et al., 2009).

### Abrogation of Glutamate-Mediated Excitotoxicity

In CNS, l-glutamate is a stimulatory neurological substance that forms memory and learning by preserving synaptic plasticity and creating neural networks (Shigeri et al., 2004; Salehi et al., 2020c). In the postsynaptic terminal, ionotropic receptors and G protein-coupled receptors come into contact with glutamate discharged from the presynaptic terminal, mediating rapid excitatory transmission (Marshall, 2001). Glutamate uptake represses the molecular action of the stimulatory amino acid regulator in glia and astrocytes accompanying the synaptic connections (Dong et al., 2009).

Excitotoxicity is associated with prolonged and downregulated stimulation of glutamate receptors, which causes epilepsy, hypoxic-ischemic brain damage, neurotrauma, and neurological diseases such as AD, MS, ALS, etc (Akyuz et al., 2021). Moreover, excitotoxicity is found worldwide via NMDA receptors mostly by the uncontrolled accumulation of Ca2+ ions (Choi, 1992), AMPA receptors or voltage-gated Ca2+ channels which trigger proteases, phospholipases, and nucleases, that provoke apoptosis and damage to the brain (Choi, 1992). Cytosolic Ca2+ homeostasis modification contributes to mitochondrial dysfunction, ROS generated stress leading toward the death of neurotoxic cells (Rego and Oliveira, 2003; Szydlowska and Tymianski, 2010; Esposito et al., 2013).

QUR from the extract of onion peels and Hypericum perforatum L, by lessening ROS Production outcome, glutamate-induced Ca2+ upsurge, retention of mitochondrial membrane potentials, and downregulation a plethora of biochemical markers linked with cell death and autophagy, neuronal cells were protected against excitotoxicity insults (Silva et al., 2008; Yang et al., 2013) (Figure 2).

### Inhibition of Cholinesterase Activity

The cholinergic hypothesis shows that the main cause of cognitive dysfunction in AD is a reduction of the number of adrenoceptors including cholinergic and acetylcholine receptors (Sabri et al., 2008). The vital enzyme implicated in controlling the synaptic level of Ach is acetylcholinesterase (AChE) (Greig et al., 2002). Several studies have shown that AChE also encourages Aβ accumulation resulting of neuronal damage in AD patients (Inestrosa et al., 2000). Therefore, AChE inhibition is considered a potential target for AD, increasing the supply of Ach in regions of the brain and reducing Aβ uptake (Anand and Singh, 2013; Colović et al., 2013). The treatment with AChE antagonists for AD patients in current symptomatic treatment is mild to moderate (Murray et al., 2013).

In AD patients, AChE inhibitors improve nicotinic receptor expression, thereby enhancing cognitive memory (Parsons et al., 2013). Additionally, AChE blockers not only amplified the synthesis of amyloid precursor protein (APP) but also attenuated Aβ toxicity (Inestrosa et al., 2000). QUR is therefore now a key component that shows AChE inhibiting activity (Islam et al., 2013). Under several *in vitro* condition, evaluation of QUR inhibitory AChE operation shown that this effect is competitive (Jung and Park, 2007). In *in vivo* PD models, elevated level QUR dosage dramatically reduces the effect of AChE in the hippocampal zone (Sriraksa et al., 2012).

### Modulation of Abnormal Protein Aggregation

In many neurodegenerative diseases, the typical pathological hallmarks are protein collapse and aggregation along with neurodegeneration (Salehi et al., 2020a). In the folding and aggregation of the protein, posttranslational changes play a pivotal part. Protein phosphorylation plays a key role in accumulation and sensitivity in most neurodegenerative disorders (Salazar and Höfer, 2009; Salehi et al., 2021b).

#### Abrogate Alpha-Synuclein Fibrillisation

Alpha-synuclein (alpha-Syn) is part of the pathological hallmark of PD as well as other diseases commonly known as synucleinopathies (Baba et al., 1998). S129, a main component of alpha-Syn, is generally associated with cytoskeletal, vesicular trafficking proteins and enzymes associated with protein serine phosphorylation (Waxman and Giasson, 2011). Multiple protein kinases such as casein kinases, G-protein coupled receptor kinases, and polo-like kinases) phosphorylation of S129 contributes to the agglomeration of alpha-Syn producing amalgamations and homeostasis of synaptic vesicles and neurotransmission defects (Saha et al., 2004). In a plethora of PD patients along with synucleinopathies including as dementia, multiple system atrophy and others, aggregate types of alpha-Syn developing amalgamations were commonly observed (Waxman and Giasson, 2008). QUR demonstrated a protective effect against synucleinopathies by effectively inhibiting alpha-Syn aggregation. QUR attaches to alpha-Syn covalently, generating QUR-alpha-Syn complex that increases hydrophilicity and prevents fibrillation. Therefore, QUR efficiently breaks down fibrils through protein-QUR interaction and separated them into stable oligomers (Zhu et al., 2013).

#### Tau Protein Alteration

Tau proteins are microtubule-associated and expressed protein in the neurons of CNS and PNS that are necessary for microtubule stabilization (Wang and Liu, 2008) (Figure 2). To determine the ability to bind to microtubules, Tau protein phosphorylation is necessary. Hyper-phosphorylation contributes to isolation, self-aggregation, and de-polymerization of the microtubule leads to final neuronal death (Tai et al., 2012). The HSP 70 and glycogen synthase kinase 3β (GSK3β) exert a critical role in tau phosphorylation and related cognitive damage in AD (Blair et al., 2013).

Updated therapeutics for attenuating tau pathologies such as drugs that inhibit tau aggregation or modify tau-effector protein activity such as kinase inhibitors (GSK3β), microtubule stabilizer and HSP70 inhibitor (Sharifi-Rad et al., 2020e). Most researchers have stated that QUR can inhibit tau pathology caused by okadaic acid by dramatically suppressing tau protein hyper-phosphorylation (Jiang et al., 2016). The inhibition of tau protein phosphorylation is made by inactivating GSK3β (Johnson et al., 2011), through the modulation of the PI3K/Akt/GSK3β pathway (Lu et al., 2006; Jiang et al., 2016).

#### Anti-Amyloidogenic Effect

Intraneuronal neurofibrillary tangles, extracellular senile plaque deposition, and vascular amyloid are found in AD (Selkoe, 2001; Sharifi-Rad et al., 2020a). Aβ peptide is formed by an aberration in APP, main sections of senile plaques, proteolytic processing, and Aβ (1–42) peptide aggregation is neurotoxic (Portelius et al., 2006). Therefore, the development of drugs that prevent the formation of amyloid or attenuate the aggregation of Aβ and facilitate the reduction of irregular fibrillary aggregates serves as an important therapeutic approach to AD care (Salloway et al., 2008).

Several *in vitro* reports demonstrate that QUR inhibits Aβ fibrils formation by enhancing the hydrophobic bond between the phenyl rings with β-sheet structures of Aβ and destabilizes the preformed mature fibrils (Ono et al., 2003; Jiménez-Aliaga et al., 2011; Zaplatic et al., 2019) (Figure 2). In addition, the B ring group 30′40-dihydroxyl plays a vital activity in the anti-aggregation effect (Jiménez-Aliaga et al., 2011).

Moreover, QUR represses Aβ peptides aggregation by promoting macroautophagy and proteasomal degradation pathways (Regitz et al., 2014). Furthermore, by direct abrogation QUR binds and inhibits β-site APP cleaving enzyme-1 (BACE-1) activity and thus prevents Aβ (1–42) formation (Shimmyo et al., 2008). Via their keto-enol group, QUR can also interact competitively with Aβ on its metal-binding site, avoiding oxidation reactive stress induced by the interaction of Aβ-Cu2+. QUR prevents amyloid aggregation through its dual inhibitory action (metal chelator and Aβ interactor) (Tay et al., 2013).

### Autophagy Induced Neuroprotective Effect

Autophagy, a cellular destructive mechanism involving the recycling of cellular components and the removal of damaged and aggregated protein by a lysosomal degradative process. The basal autophagy process is the key functional integral component of the CNS, preventing damaged components and protein consumption in the nervous system through its defensive protocol (Siokas et al., 2021).

Mutations contribute to neurodegeneration via amplifying the gene-related to autophagic processes (Nixon, 2013). Reports have shown that QUR serves as an efficient autophagy simulator in high glucose Schwann cells (Qu et al., 2014), and QUR antagonizes Aβ (1–42) mediated neurotoxicity by inducing autophagy processes (Regitz et al., 2014).

### Quercetin and Neurotrophic Factors

For the development of a functionally improved nervous system, there are neurite formation and synaptic patterning (Nussbaum et al., 2017). As the injured brain which an absence of regenerative ability, natural bioactive phytochemicals can improve the action of the neurotrophic factors that help to promote neurite outgrowth, contributing to neurological regeneration (Moore et al., 2006). *In vitro* studies showed that QUR and its derivative (isoquercetin) induced substantial neurotrophin (NGF) and BDNF-induced neurite outgrowth through activation of Na+/K+/2Cl-cotransporter isoform (NKCC1) (Nakajima et al., 2011) and modulate Rho GTPase enzyme activity (Palazzolo et al., 2012).

QUR significantly induces neuritis and neurite duration at a lower concentration (1 nM) by triggering the P13K/AKT pathway (Tangsaengvit et al., 2013). QUR has increased neurotrophic neurite-related proteins (e.g., growth-related protein-43 expression, microtubule-related protein (MAP) and tau, synaptophysin and synapsin) and promotes neuronal survival (Moosavi et al., 2016). In addition, QUR showed improved brain development and synapses through CREB (cyclic AMP response element-binding protein) phosphorylation which important for growth of neurite, thus increasing the rate of a neurotrophic factor generated from the brain that is essential for neurogenesis (Tchantchou et al., 2009). Table 1 summarized the neuroprotective effects of QUR in several *in vitro* and *in vivo* models.

**TABLE 1:** Neuroprotective effects of quercetin and/or its derivatives against various neurodegenerative diseases and other brain disorders.

Quercetin/Derivatives/Source	Test model *in vitro/in vivo*	Exposure	Effects/Molecular mechanisms	References
**Alzheimer’s disease**				
Quercetin	HT-22 mouse hippocampal cells/*in vitro*	Glutamate induced toxicity	↓lipid peroxidation, ↓GSH oxidation, ↓ROS	Ishige et al. (2001)
	HEK 293 human embryonic kidney cells/*in vitro*	Aβ (1–42)	↓ Aβ peptides, ↓the performed mature fibrils	Ono et al. (2003)
	HT22 murine neuroblastoma cells/*in vitro*	Aβ (25–35)	↓ amyloidogenic Aβ peptides	Kim et al. (2005)
	Primary hippocampal cultures/*in vitro*	Aβ (1–42)	↓apoptosis, ↓ ROS, ↓mediated damage	Ansari et al. (2009)
Quercetin/*Ginkgo biloba*	SHSY5Y human neuroblastoma cells/*in vitro*	Aβ (1–42)	↓Akt signaling pathways, ↓ERK1/2, ↓JNK, ↓Aβ toxicity, ↓platelet-activating factor	Shi et al. (2009)
Quercetin-3′-glucoside	PC12 cells/*in vitro*	Aβ	↓H_2_O_2_ ↑CREB/BDNF signaling pathway, ↓Aβ, ↓ROS	Yu et al. (2020)
Quercetin/ginkgoflavonols	Double Transgenic (TgAPP/PS1) mice/*in vivo*	-	Reversed the spatial learning deficit	Hou et al. (2010)
Quercetin	APP stable cells/*in vitro*	Aβ (25–35)	↓ROS, ↓BACE, ↓ Aβ, ↓GSH, ↓lipid peroxidation	Jiménez-Aliaga et al. (2011)
Quercetin-3-*O* glucuronide	APP695-transfected SH-SY5Y cells/*in vitro* Tg2576 AD primary neuron cultures/*in vitro*	Aβ (1–42)	↓Aβ peptides, ↑CREB signaling, ↓Aβ aggregation, ↑mitogen-activated protein kinase, ↑neuronal survival, ↑c-Jun N-terminal kinases, ↓stress-induced impairments	Ho et al. (2013)
Quercetin/*Acanthopanax henr*	Cell free system/*in vitro*	-	↓ acetylcholinesterase, ↑antioxidant activity	Zhang et al. (2014)
Quercetin	Triple-transgenic mouse model of AD/*in vivo*	-	↓tauopathy, ↓β-amyloidosis, ↑memory, ↑learning ↓microgliosis, ↓astrogliosis	Sabogal-Guáqueta et al. (2015)
	APP23 AD model mice/*in vivo*	-	↓eIF2α, ↓ATF4, ↓GADD34, ↑memory in aged mice, ↓deterioration in memory at the early stage of AD	Hayakawa et al. (2015)
**Parkinson’s disease**				
Quercetin	Microglial (N9)-neuronal (PC12) cells/*in vitro*	MPP	↓iNOS in microglial cells, ↓DNA fragmentation, ↑apoptosis, ↓nuclear translocation of apoptosis-inducing factor, ↓caspase-3 activation	Bournival et al. (2012)
Quercetin glycoside	PC12 cells/*in vitro*	6-OHDA	↑antioxidant activity, ↑GSH, ↑GPx	Magalingam et al. (2016)
Quercetin	Wistar rats/*in vivo*	6-OHDA	↑spatial memory, ↓oxidative stress, ↓AChE activity, ↑antioxidant activity, ↓neuronal damage	Sriraksa et al. (2012)
Quercetin	Mice/*in vivo*	MPTP	↓striatal dopamine depletion, ↓motor deficits, ↑GPx, ↑SOD	Lv et al. (2012)
Quercetin	Cell-free system	α-Synuclein	↓Aβ fibrillation	Zhu et al. (2013)
Quercetin	Wistar rats/*in vivo*	Roteno	↓nigral GSH depletion, ↓ROS, ↓striatal DA loss, ↑mitochondrial complex, ↑activity and scavenging hydroxyl radical, ↓ neuronal death	Karuppagounder et al. (2013)
Isoquercetin	PC12 cells/*in vitro*	6-OHDA	↓ROS, ↑SOD, ↑ antioxidant enzymes (GSH, catalase, glutathione peroxidase)	Magalingam et al. (2014)
Quercetin	Wistar rats/*in vivo*	Haloperidol MPTP	↑the cataleptic score, ↓actophotometer activity, ↑GSH, ↓lipid peroxidation, ↓ROS	Pany et al. (2014)
Quercetin + fish oil	Wistar rats/*in vivo*	Rotenone	↑mitochondrial functions, ↑GSH, ↑antioxidant defences	Denny Joseph and Muralidhara (2013)
**Hutington’s disease**				
Quercetin	Wistar rats/*in vivo*	3-NP	↑ATP, ↑activity of complex II and V enzyme of respiratory chain complex, ↓ROS, ↑SOD, ↑catalase, ↓lipid peroxidation	Sandhir and Mehrotra (2013)
Quercetin + fish oil	Wistar rats/*in vivo*	3-NP	↓oxidative stress, ↑motor function	Denny Joseph and Muralidhara (2013)
Quercetin + sesamol	Wistar rats/*in vivo*	QA	↓neurochemical alterations in the rat brain, ↓behavioral, ↓biochemical, ↑antioxidant effects, ↑ anti-inflammatory activity	Kuhad et al. (2013)
Quercetin + lycopene	Wistar rats/*in vivo*	3-NP	↓anxiety, ↓depression	Jain and Gangshettiwar (2014)
Quercetin	Sprague dawley rats/*in vivo*	3-NP	↓gait despair, ↓microglial proliferation, ↓anxiety, ↑astrocyte numbers in the lesion core, ↓motor coordination deficits, ↓serotonin metabolism	Chakraborty et al. (2014)
**Multiple sclerosis**				
Quercetin	Human umbilical cord blood-derived cultured mast cells/*in vitro*	IL-1	↓demyelination, ↑PKC phosphorylation, ↑IL-6, ↓IL-1, ↑p38, ↓mast cell activation	Kandere-Grzybowska et al. (2006)
Quercetin	Peripheral blood mononuclear cells isolated from MS patients/*in vitro*	-	↑IL-6, ↓immune response, ↓TNF-α, ↓demyelination	Sternberg et al. (2008)
**Brain ischemic injury**				
Quercetin	Sprague- dawley rats/*in vivo*	Traumatic brain injury	↓neutrophil infiltration, ↑GSH, ↓myeloperoxidase activity	Graham et al. (2000)
Quercetin	Sprague- dawley rats/*in vivo*	Acute traumatic spinal cord injury	↑iron clearance in the spinal cord through its ↑chelating effect, ↑motor function	Schultke et al. (2010)
Quercetin	Swiss albino mice/*in vivo*	High altitude hypoxia	↓HIF1α, ↓hypoxia, ↓VEGF, ↓brain dysfunction, ↓active caspase 3, ↓ubiquitin	Sarkar et al. (2012)
Quercetin-3-O methyl ether	Sprague- dawley rats/*in vivo*	MCAO rat model	↓ oedema, ↓oxidative stress-mediated damage, ↓behavioral deficit	Jung et al. (2012), Lee et al. (2015)
Quercetin	Sprague– Dawley rats/*in vivo*	MCAO rat model	↑PI3K/Akt, ↑antioxidative, ↑anti-apoptotic signaling	Chang et al. (2014)
Quercetin	Wistar rats/*in vivo*	Global cerebral ischemia	↑AKT, ↑anti-apoptotic signaling pathway, ↑antioxidant activity, ↓ROS	Lei et al. (2015)
Quercetin	Sprague-dawley rats/*in vivo*	Transient focal cerebral ischemia	↑Ca^2+^ into the mitochondrial matrix, ↑electron transport chain activity	Nichols et al. (2015)
Quercetin + vitamin E	Primary cortical neurons/*in vitro*	Induced ischemic stroke	↑CREB phosphorylation, ↑ NO, ↑autophosphorylation of CaMK II, IV, ↑Ca^2+^/calmodulin-dependent kinases II, IV, ↑mitochondrial biogenesis, ↑ BCl-2	Nichols et al. (2015)
**Epilepsy**				
Quercetin/*Anisomeles malabarica*	Wistar rats/*in vivo*	Diazepam + PTZ	Stimulating GABA_A_ receptors, NMDA receptors’ antagonist	Choudhary et al. (2011)
Quercetin	Wistar rats/*in vivo*	PTZ	↑anticonvulsant effects, ↓seizure severity, ↓lipid peroxidation via its ↑antioxidant effect, ↑memory retrieval in the passive avoidance task	Nassiri-Asl et al. (2013)
Quercetin	Albino rats/*in vivo*	PTZ	↑ antiseizure effect, ↑anticonvulsant effect	Sefil et al. (2014)
Quercetin	Wistar rats/*in vivo*	6-OHDA	↓excitability in neurons involved in epilepsy,↓neuroplastic changes in neural circuits, ↓NMDA receptor functionality	Mehdizadeh et al. (2009)
**Miscellaneous neurotoxin**				
Quercetin,/*Opuntia ficus-indica*	Rat cortical cells/*in vitro*	H_2_O_2_; xanthine/xanthine oxide	↑antioxidant activity	Dok-Go et al. (2003)
Quercetin	Mice/*in vivo*	Ethanol intoxication	↓cognitive impairment, ↑antioxidant mechanisms	Singh et al. (2003)
Quercetin	SH-SY5Y neuroblastoma cells/*in vitro*	6-OHDA	↑antioxidant activity	Kaariainen et al. (2008)
Quercetin/*Ginkgo biloba*	N2a cells/*in vitro*	Juglone (5- hydroxy-1, 4-napthoquinone)	↓ROS	Smith and Luo (2003)
Quercetin-3-O-galactoside)/*Hypericum perforatum*	PC12 cells/*in vitro*	H_2_O_2_/tert-butyl hydroperoxide	↓apoptosis mediated cell death, ↓ROS, chelates the transition metal ions	Liu et al. (2005)
Quercetin-3-O-β-d-glucopyranoside/*Echinophora cinerea*	PC12 cells/*in vitro*	H_2_O_2_	↓ ROS	Shokoohinia et al. (2015)
Quercetin	PC12 cells/*in vitro*	H_2_O_2_/Xanthine oxidase	↓neuronal injury (IC_50_ = 0.5–0.7 μg/ml), ↑antioxidative effect	Shokoohinia et al. (2015)
Quercetin	SH-SY5Y cells/*in vitro*	H_2_O_2_	↓pro-apoptotic bax gene, ↓lactate dehydrogenase, ↑antiapoptotic Bcl-2, ↓caspase cascade, ↑DNA fragmentation, ↑apoptosis	Suematsu et al. (2011)
Quercetin	ICR mice/*in vivo*	Trimethyltin	↓acetylcholinesterase; ↓peroxidation of polyunsaturated fatty acid in membrane, ↑cognitive ability	Choi et al. (2012)
Quercetin	Mice/*in vivo*	High cholesterol-induced neurotoxicity	↑AMPK, ↓activation of microglia, ↓iNOS, ↓COX-2, ↓IL-1β, ↓TNF-α, ↓BACE1	Lu et al. (2010)
Quercetin	Wistar rats/*in vivo*	Cadmium intoxication	↓AChE, ↓NTPDase, ↓ADA activities in cerebral cortex synaptosomes	Abdalla et al. (2013)
Quercetin	Swiss Albino mice/*in vivo*	Olfactory bulbectomy	↓NMDA receptors, ↑NO, ↓depression, ↑antioxidant activity	Holzmann et al. (2015)
Quercetin	Chinese kunming mice/*in vivo*	High-fat diet	↑HDL decrease, ↓ total cholesterol, ↑CREB, ↓oxidative damage, ↑PI3 K/AKT/Nrf2, ↓ROS, ↓MDA, ↑cognitive impairment	Xia et al. (2015)
3‘-O-(3-chloropivaloyl) quercetin (CPQ)	BV-2 microglial cells/*in vitro*	Lipopolysaccharides	↓NF-κB, ↓inflammatory mediators: ↓NO, ↓TNF-α, ↓iNOS; ↓ proliferation of BV-2 microglial cells	Mrvova et al. (2015)
Quercetin	Albino rats/*in vivo*	Aluminum	↓mitochondrial DNA oxidation, ↓ROS, ↓oxidative stress, ↑Bcl-2,, ↓p53, ↑MnSOD, ↓translocation of cytochrome-c, ↓Bax, ↓caspase-3, ↓DNA damage	Sharma et al. (2016)
**Aging and cognitive function**				
Quercetin	Kunming mice/*in vivo*	Galactose	↑SOD, ↑cognitive impairment, maintain Ca^2+^ homeostasis, ↑GAP43 mRNA expression, ↑normal function of neurons	Lu et al. (2006)
Quercetin	*Caenorhabditis elegans/in vivo*	Thermal stress	↑radical scavenging activity, ↓MnSOD	Saul et al. (2008)
Quercetin/Quercetin caprylate	HFL-1 primary human fibroblasts/*in vitro*	-	↑cellular lifespan, ↑proteasome activation, ↑neuronal survival, ↑antiaging effect; ↑rejuvenating effect, ↑antioxidant properties	Chondrogianni et al. (2010)

### Targeting of Sirtuins in Age-Related Neurodegenerative Disorders

Sirtuins (SIRT 1–7), a member of signal proteins that exert a role in metabolic regulation, stress and longevity responses. SIRT1 is predominantly located in the hippocampus of the brain. A few studies showed that the induction of SIRT1 by calorie restriction (CR) diet determine the protective effect against dopamine receptor neurodegenerative disorder (Tchantchou et al., 2009; Gräff et al., 2013), attenuating Aβ peptide formation, and enhancing longevity (Nazir and Jadiya, 2013).

SIRT-1 is responsible for Aβ peptide inhibition, Bax-induced apoptosis suppression and suppression of a plethora of other pro-apoptotic factors (Aloizou et al., 2021). Natural bioactive compounds or drugs may act as neuroprotective agents by modulation of SIRT1 protein expression (Salehi et al., 2021a). QUR has been reported to exhibit neuroprotective effect and anti-ageing effect via induction and activation SIRT1 (de Boer et al., 2006) (Figure 2). Studies indicate that QUR can activate SIRT1-dependent pathways and modulate pro-inflammatory substances, indicating its use to treat MS and ALS (Hendriks et al., 2003).

### Memory Enhancement Effect

In various *in situ* models, QUR has been documented to penetrate the blood-brain barrier (Youdim et al., 2004), which may be one of the competing variables for its cognitive enhancement ability. Many studies have shown that in several neurodegenerative disorders, including PA, AD and chronic cerebral ischemia models, QUR can alleviate behavioral and cognitive disability (Yao et al., 2010). QUR improves learning and memory in the AD animal model, decreases senile plaques, mitochondrial dysfunction and increases the antioxidative mechanism through AMP protein kinase, and can enhance cognitive deficits (Wang et al., 2014).

QUR has oxidative defense and anti-apoptotic activity and enhanced memory dysfunction caused by hypoxia (Prasad et al., 2013; Boyina et al., 2020). By suppressing oxidative stress, QUR improves cognitive deficit (Mohammadi et al., 2014). QUR has also increased learning and memory problems (Kumar et al., 2008; Richetti et al., 2011; Sharma et al., 2013). In addition, by increasing GSH level, scavenge hydroxyl free radical, Na+/K+ adenosine triphosphatase and suppressing iNOS activity, QUR attenuated cognitive impairments and neurological changes in the brain of mice (Sun et al., 2007).

In addition, QUR strengthened AD’s spatial memory through antioxidant and scavenging properties (Ashrafpour et al., 2015). In mice, QUR supplements administered showed improved learning and memory function (Liu et al., 2006).

## Discussion

The most serious health conditions in the modern period are known to be brain disorders (Salehi et al., 2019b). More than 600 diseases affect the CNS, including the 21st century scourge of neurodegenerative conditions includes AD, ALS, PD, and HD (Magalingam et al., 2018; Sharifi-Rad et al., 2020c). Due to the differences in signs involved with any of these CNS diseases, the basic mechanisms of neurodegeneration are interrelated (Calina et al., 2020). Oxidative damage, mitochondrial malfunction, glutamate excitotoxicity, inflammatory reaction, protein accumulation and modification in metal ion homeostasis are the principal factors leading to CNS disorders (Chen et al., 2005; Nieoullon, 2011; Rasool et al., 2014).

**FIGURE 3:**
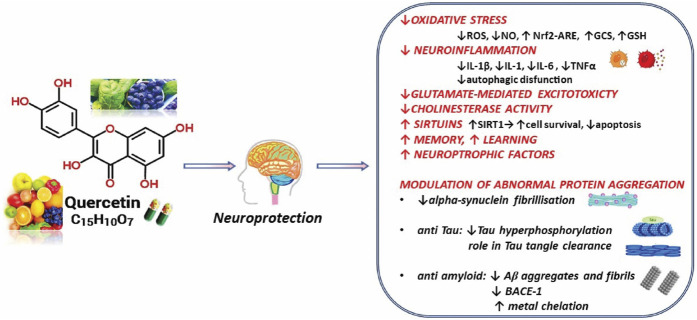
Summarized scheme with relevant neuroprotective effects of Quercetin. *Abbreviations:* ↑, increase; ↓, decrease; Aβ, amyloid beta-peptide; BACE1, beta-secretase 1; GCS, γ-glutamyl-cysteine synthetase; GSH, glutathione; IL, interleukin; NO, nitric oxide; Nrf2-ARE, Nuclear factor erythroid-derived 2-like 2- antioxidant responsive element; ROS, reactive oxygen species; SIRT1, sirtuin 1; TNF-α, tumor necrosis factor-alfa.

In the current analysis, the multitargeted molecular mechanisms highlighted in preclinical studies have been outlined concerning QUR and its potential neuroprotective impact (Figure 3).

A strong point of this paper is that many studies have been included with recent meta-analyses and reviews that contain the most important data synthesized by the mechanisms of action and molecular targets of Quercetin on its neuroprotective effect.

Through this ability to modify the functioning of chemical synapses, QUR has an important role in neural plasticity. Plasticity is a key factor in the neuronal development of the brain, the proper functioning of the nervous system, adaptation to the changing environment, aging and other chronic diseases (Barreca et al., 2016).

The main therapeutic limitation derives from the fact that studies have shown that the absorption of QUR through food is quite low. Also due to reduced oral bioavailability, QUR should be administered parenterally to exert its neuroprotective effects. New therapeutic horizons were opened with obtaining recent nano-pharmaceutical formulations such as micelles, phytosomes or encapsulated QUR, that increase its brain concentration. (Benameur et al., 2021).

The QUR’s activity in food depends on the method of preparation, harvest time and storage conditions. The absorption of QUR is enhanced by the presence of dietary fats (being a lipophilic compound), and the presence of insoluble fibers. Bioavailability is better if whole foods are consumed (Dabeek and Marra, 2019). Although QUR is present in many foods, the diet covers between 0 and 30 mg of QUR per day (Batiha et al., 2020). No clear studies are indicating the daily requirement, but it is assumed that this dose is well below our needs.

QUR is a compound that is part of bioflavonoids, a potent anti-oxidative and anti-inflammatory natural compound found in several vegetables and fruits (Chen et al., 2020; Sharifi-Rad et al., 2020d). Due to its anti-inflammatory effect, some studies have shown that QUR can be useful as a complementary treatment for neurodegenerative diseases such as AD or PD, by stimulating the expression of HO-1, which reduces NO production and suppress pro-inflammatory markers, TNF-α and IL-1α. QUR also protects the brain from the toxicity associated with d-galactose. This protection is associated with QUR's ability to elevate superoxide dismutase (SOD) activity and downturn malondialdehyde (MDA) levels. These antioxidant properties of QUR can also be useful in mitigation of the normal aging of the brain when oxidative stress increases through multiple mechanisms such as lipid peroxidation, protein oxidation and the release of ROS from mitochondria (Elumalai and Lakshmi, 2016).

Since it is a food-derived biomolecule, QUR seems to be safe and has low risks. The recommended dietary allowance approved by the FDA as GRAS (Generally Accepted as Safe) for pure QUR is 1.5 g/day (FDA, 2010). However, interaction with other drugs is possible. Co-administration of QUR with some antibiotics can reduce antibiotic efficacy and, owing to its antiplatelet and anticoagulant properties, it is not recommended that antibiotics be used concurrently with other antithrombotics. Among QUR's pharmacodynamic interactions with other medications, those with blood clotting effects are the most important. There are no known associations with food, other plants or other supplements. Taken together, these studies demonstrate that dietary QUR supplementation can serve as a potential candidate in the prevention of neurological and neurodegenerative conditions at minimum dose levels.

## Conclusion Remarks and Future Perspectives

To conclude, QUR serves as an effective therapeutic agent against various neurological disorders by reducing stress, inflammatory response and fostering brain growth. The overall analysis highlights the various neuroprotection aspects of QUR. Therefore, QUR can be considered as a strong dietary supplement with minimal toxicity as part of plant matrices. While QUR demonstrates pluripotent neuroprotective effects due to its poor solubility, bioavailability and instability in different *in vitro* and *in vivo* degenerative experimental models, its application in the pharmaceutical sector is restricted. Scientific data mostly on clinical studies and toxicity of QUR is also negligible.

Therefore, a need for more study to concentrate firstly on drug delivery mechanisms such as prodrugs, nanoencapsulation and microemulsion to enhance bioavailability and permeability of the blood-brain; 2) more clinical trials to evaluate the effective dose for the treatment of neurodegenerative disorders; 3) further study of the distribution of QUR metabolites in the experimental mod CNS; 4) To assess the neurotoxic effect, up-to-date profiling of *in vivo* toxicity.

## Abbreviations

↑, increased; ↓, decreased; 3-NP, 3-nitropropionic acid; 6-OHDA, 6-hydroxy dopamine; ADA, adenosine deaminase; AChE, acetylcholinesterase; AKT, protein kinase B; AMPK, AMP-activated protein kinase; ALP, alkaline phosphatase; BACE1, beta-secretase 1; Bax Bcl-2, associated X; Bcl-2B-cell lymphoma 2; COX-2, Cyclooxygenase-2; CREB, cAMP response element-binding protein; GABA, γ-aminobutyric acid; GSH, glutathione; GT, glutamyl transpeptidase; hBMECs, brain microvascular endothelial cells; HIF1α, hypoxia inducible factor 1α; IL, interleukin-1; iNOS, inducible nitric oxide synthase; MCAO, middle cerebral artery occlusion; MPP + 1-methyl-4- phenylpyridinium ion; MPTP 1-methyl-4- phenyl-1, 2,3,6-tetrahydropyridine; NF-κB, nuclear factor kappa B; NO, nitric oxide; NTPDase nucletoidase nucleoside triphosphate diphosphohydrolase; PI3K, phosphatidylinositol 3 kinase; PTZ, pentylenetetrazole; QA, quinolinic acid; SOD, superoxide dismutase; ROS, reactive oxygen species; TNF-α tumor necrosis factor-alfa; VEGF, vascular endothelial growth factor.

## References

[B1] Abdalla F. H. Cardoso A. M. Pereira L. B. Schmatz R. Gonçalves J. F. Stefanello N. (2013). Neuroprotective Effect of Quercetin in Ectoenzymes and Acetylcholinesterase Activities in Cerebral Cortex Synaptosomes of Cadmium-Exposed Rats. Mol. Cel Biochem 381 (1-2), 1–8. 10.1007/s11010-013-1659-x 23797318

[B2] Abdalla F. H. Schmatz R. Cardoso A. M. Carvalho F. B. Baldissarelli J. de Oliveira J. S. (2014). Quercetin Protects the Impairment of Memory and Anxiogenic-like Behavior in Rats Exposed to Cadmium: Possible Involvement of the Acetylcholinesterase and Na+,K+-ATPase Activities. Physiol. Behav. 135, 152–167. 10.1016/j.physbeh.2014.06.008 24952260

[B3] Ader P. Wessmann A. Wolffram S. (2000). Bioavailability and Metabolism of the Flavonol Quercetin in the Pig. Free Radic. Biol. Med. 28 (7), 1056–1067. 10.1016/s0891-5849(00)00195-7 10832067

[B4] Akyuz E. Paudel Y. N. Polat A. K. Dundar H. E. Angelopoulou E. (2021). Enlightening the Neuroprotective Effect of Quercetin in Epilepsy: From Mechanism to Therapeutic Opportunities. Epilepsy Behav. 115, 107701. 10.1016/j.yebeh.2020.107701 33412369

[B5] Alam Q. Zubair Alam M. Mushtaq G. A. Damanhouri G. Rasool M. Amjad Kamal M. (2016). Inflammatory Process in Alzheimer's and Parkinson's Diseases: Central Role of Cytokines. Cpd 22 (5), 541–548. 10.2174/1381612822666151125000300 26601965

[B6] Aloizou A.-M. Siokas V. Pateraki G. Liampas I. Bakirtzis C. Tsouris Z. (2021). Thinking outside the Ischemia Box: Advancements in the Use of Multiple Sclerosis Drugs in Ischemic Stroke. Jcm 10 (4), 630. 10.3390/jcm10040630 33562264 PMC7914575

[B7] Alvarez-Arellano L. Salazar-García M. Corona J. C. (2020). Neuroprotective Effects of Quercetin in Pediatric Neurological Diseases. Molecules 25 (23), 5597. 10.3390/molecules25235597 PMC7731313 33260783

[B8] Anand P. Singh B. (2013). A Review on Cholinesterase Inhibitors for Alzheimer's Disease. Arch. Pharm. Res. 36 (4), 375–399. 10.1007/s12272-013-0036-3 23435942

[B9] Ansari M. A. Abdul H. M. Joshi G. Opii W. O. Butterfield D. A. (2009). Protective Effect of Quercetin in Primary Neurons against Aβ(1-42): Relevance to Alzheimer's Disease. J. Nutr. Biochem. 20 (4), 269–275. 10.1016/j.jnutbio.2008.03.002 18602817 PMC2737260

[B10] Arredondo F. Echeverry C. Abin-Carriquiry J. A. Blasina F. Antúnez K. Jones D. P. (2010). After Cellular Internalization, Quercetin Causes Nrf2 Nuclear Translocation, Increases Glutathione Levels, and Prevents Neuronal Death against an Oxidative Insult. Free Radic. Biol. Med. 49 (5), 738–747. 10.1016/j.freeradbiomed.2010.05.020 20554019

[B11] Ashrafpour M. Parsaei S. Sepehri H. (2015). Quercetin Improved Spatial Memory Dysfunctions in Rat Model of Intracerebroventricular Streptozotocin-Induced Sporadic Alzheimer's Disease. Natl. J. Physiol. Pharm. Pharmacol. 5, 411–415. 10.5455/njppp.2015.5.2308201563

[B12] Assi A. A. El Sayed A. M. (1998). Evaluation of the Anti-inflammatory Profile of Quercetin Enhancing its Effects by Beta-Cyclodextrin. J. Drug Res. Egypt. 22, 293–320.

[B13] Baba M. Nakajo S. Tu P. H. Tomita T. Nakaya K. Lee V. M. (1998). Aggregation of Alpha-Synuclein in Lewy Bodies of Sporadic Parkinson's Disease and Dementia with Lewy Bodies. Am. J. Pathol. 152 (4), 879–884. 9546347 PMC1858234

[B14] Barreca D. Bellocco E. D';Onofrio G. Fazel Nabavi S. Daglia M. Rastrelli L. (2016). Neuroprotective Effects of Quercetin: From Chemistry to Medicine. Cnsnddt 15 (8), 964–975. 10.2174/1871527315666160813175406 27528470

[B15] Batiha G. E.-S. Beshbishy A. M. Ikram M. Mulla Z. S. El-Hack M. E. A. Taha A. E. (2020). The Pharmacological Activity, Biochemical Properties, and Pharmacokinetics of the Major Natural Polyphenolic Flavonoid: Quercetin. Foods 9 (3), 374. 10.3390/foods9030374 PMC7143931 32210182

[B16] Benameur T. Soleti R. Porro C. (2021). The Potential Neuroprotective Role of Free and Encapsulated Quercetin Mediated by miRNA against Neurological Diseases. Nutrients 13 (4), 1318. 10.3390/nu13041318 33923599 PMC8073422

[B17] Blair D. R. Lyttle C. S. Mortensen J. M. Bearden C. F. Jensen A. B. Khiabanian H. (2013). A Nondegenerate Code of Deleterious Variants in Mendelian Loci Contributes to Complex Disease Risk. Cell 155 (1), 70–80. 10.1016/j.cell.2013.08.030 24074861 PMC3844554

[B18] Boesch-Saadatmandi C. Pospissil R. Graeser A.-C. Canali R. Boomgaarden I. Doering F. (2009). Effect of Quercetin on Paraoxonase 2 Levels in RAW264.7 Macrophages and in Human Monocytes-Role of Quercetin Metabolism. Ijms 10 (9), 4168–4177. 10.3390/ijms10094168 19865538 PMC2769159

[B19] Bournival J. Plouffe M. Renaud J. Provencher C. Martinoli M.-G. (2012). Quercetin and Sesamin Protect Dopaminergic Cells from MPP+-induced Neuroinflammation in a Microglial (N9)-Neuronal (PC12) Coculture System. Oxidative Med. Cell Longevity 2012, 1–11. 10.1155/2012/921941 PMC3418684 22919443

[B20] Boyina H. K. Geethakhrishnan S. L. Panuganti S. Gangarapu K. Devarakonda K. P. Bakshi V. (2020). In Silico and *In Vivo* Studies on Quercetin as Potential Anti-parkinson Agent. Adv. Exp. Med. Biol. 1195, 1–11. 10.1007/978-3-030-32633-3_1 32468451

[B21] Buga A.-M. Docea A. Albu C. Malin R. Branisteanu D. Ianosi G. (2019). Molecular and Cellular Stratagem of Brain Metastases Associated with Melanoma (Review). Oncol. Lett. 17 (5), 4170–4175. 10.3892/ol.2019.9933 30944612 PMC6444343

[B22] Bureau G. Longpré F. Martinoli M.-G. (2008). Resveratrol and Quercetin, Two Natural Polyphenols, Reduce Apoptotic Neuronal Cell Death Induced by Neuroinflammation. J. Neurosci. Res. 86 (2), 403–410. 10.1002/jnr.21503 17929310

[B23] Burlec A. F. Macovei I. Sacarescu A. Corciova A. Mircea C. Iancu C. E. (2020). ESSENTIAL OILS IN WELLNESS CENTERS: OVERVIEW ON EUROPEAN UNION LEGISLATION, POTENTIAL THERAPEUTIC EFFECTS AND TOXICITY. Farmacia 68 (6), 992–998. 10.31925/farmacia.2020.6.5

[B24] Calina D. Buga A. M. Mitroi M. Buha A. Caruntu C. Scheau C. (2020). The Treatment of Cognitive, Behavioural and Motor Impairments from Brain Injury and Neurodegenerative Diseases through Cannabinoid System Modulation-Evidence from *In Vivo* Studies. Jcm 9 (8), 2395. 10.3390/jcm9082395 PMC7464236 32726998

[B25] Chakraborty J. Singh R. Dutta D. Naskar A. Rajamma U. Mohanakumar K. P. (2014). Quercetin Improves Behavioral Deficiencies, Restores Astrocytes and Microglia, and Reduces Serotonin Metabolism in 3-nitropropionic Acid-Induced Rat Model of Huntington's Disease. CNS Neurosci. Ther. 20 (1), 10–19. 10.1111/cns.12189 24188794 PMC6493046

[B26] Chang H.-C. Yang Y.-R. Wang P. S. Wang R.-Y. (2014). Quercetin Enhances Exercise-Mediated Neuroprotective Effects in Brain Ischemic Rats. Med. Sci. Sports Exerc. 46 (10), 1908–1916. 10.1249/mss.0000000000000310 24561812

[B27] Chaudhary S. Ganjoo P. Raiusddin S. Parvez S. (2015). Nephroprotective Activities of Quercetin with Potential Relevance to Oxidative Stress Induced by Valproic Acid. Protoplasma 252 (1), 209–217. 10.1007/s00709-014-0670-8 25000991

[B28] Chen J.-C. Ho F.-M. Pei-Dawn Lee Chao C. Chen C.-P. Jeng K.-C. G. Hsu H.-B. (2005). Inhibition of iNOS Gene Expression by Quercetin Is Mediated by the Inhibition of IκB Kinase, Nuclear Factor-Kappa B and STAT1, and Depends on Heme Oxygenase-1 Induction in Mouse BV-2 Microglia. Eur. J. Pharmacol. 521 (1-3), 9–20. 10.1016/j.ejphar.2005.08.005 16171798

[B29] Chen L. Shen T. Zhang C. P. Xu B. L. Qiu Y. Y. Xie X. Y. (2020). Quercetin and Isoquercitrin Inhibiting Hepatic Gluconeogenesis through Lkb1-Ampka Pathway. Acta Endo (Buc) 16 (1), 9–14. 10.4183/aeb.2020.9 PMC7363997 32685032

[B30] Chen T.-J. Jeng J.-Y. Lin C.-W. Wu C.-Y. Chen Y.-C. (2006). Quercetin Inhibition of ROS-dependent and -independent Apoptosis in Rat Glioma C6 Cells. Toxicology 223 (1-2), 113–126. 10.1016/j.tox.2006.03.007 16647178

[B31] Choi D. W. (1992). Excitotoxic Cell Death. J. Neurobiol. 23 (9), 1261–1276. 10.1002/neu.480230915 1361523

[B32] Choi G. N. Kim J. H. Kwak J. H. Jeong C.-H. Jeong H. R. Lee U. (2012). Effect of Quercetin on Learning and Memory Performance in ICR Mice under Neurotoxic Trimethyltin Exposure. Food Chem. 132 (2), 1019–1024. 10.1016/j.foodchem.2011.11.089

[B33] Chondrogianni N. Kapeta S. Chinou I. Vassilatou K. Papassideri I. Gonos E. S. (2010). Anti-ageing and Rejuvenating Effects of Quercetin. Exp. Gerontol. 45 (10), 763–771. 10.1016/j.exger.2010.07.001 20619334

[B34] Choudhary N. Bijjem K. R. V. Kalia A. N. (2011). Antiepileptic Potential of Flavonoids Fraction from the Leaves of Anisomeles Malabarica. J. Ethnopharmacology 135 (2), 238–242. 10.1016/j.jep.2011.02.019 21354295

[B35] Colovic M. B. Krstic D. Z. Lazarevic-Pasti T. D. Bondzic A. M. Vasic V. M. (2013). Acetylcholinesterase Inhibitors: Pharmacology and Toxicology. Cn 11 (3), 315–335. 10.2174/1570159x11311030006 PMC3648782 24179466

[B36] Costa L. G. de Laat R. Dao K. Pellacani C. Cole T. B. Furlong C. E. (2014). Paraoxonase-2 (PON2) in Brain and its Potential Role in Neuroprotection. Neurotoxicology 43, 3–9. 10.1016/j.neuro.2013.08.011 24012887 PMC3942372

[B37] Dabeek W. M. Marra M. V. (2019). Dietary Quercetin and Kaempferol: Bioavailability and Potential Cardiovascular-Related Bioactivity in Humans. Nutrients 11 (10), 2288. 10.3390/nu11102288 PMC6835347 31557798

[B38] de Boer V. C. J. de Goffau M. C. Arts I. C. W. Hollman P. C. H. Keijer J. (2006). SIRT1 Stimulation by Polyphenols Is Affected by Their Stability and Metabolism. Mech. Ageing Development 127 (7), 618–627. 10.1016/j.mad.2006.02.007 16603228

[B39] del Rio M. A. Sanchez-Reus M. I. Iglesias I. Pozo M. A. Garcia-Arencibia M. Fernandez-Ruiz J. (2013). Neuroprotective Properties of Standardized Extracts of *Hypericum perforatum* on Rotenone Model of Parkinson's Disease. Cnsnddt 12 (5), 665–679. 10.2174/1871527311312050013 23469842

[B40] Denny Joseph K. M. Muralidhara (2013). Enhanced Neuroprotective Effect of Fish Oil in Combination with Quercetin against 3‐nitropropionic Acid Induced Oxidative Stress in Rat Brain. Prog. Neuro-Psychopharmacology Biol. Psychiatry 40, 83–92. 10.1016/j.pnpbp.2012.08.018 22960609

[B41] Dok-Go H. Lee K. H. Kim H. J. Lee E. H. Lee J. Song Y. S. (2003). Neuroprotective Effects of Antioxidative Flavonoids, Quercetin, (+)-dihydroquercetin and Quercetin 3-methyl Ether, Isolated from Opuntia Ficus-Indica Var. Saboten. Brain Res. 965 (1-2), 130–136. 10.1016/s0006-8993(02)04150-1 12591129

[B42] Dong X.-x. Wang Y. Qin Z.-h. (2009). Molecular Mechanisms of Excitotoxicity and Their Relevance to Pathogenesis of Neurodegenerative Diseases. Acta Pharmacol. Sin 30 (4), 379–387. 10.1038/aps.2009.24 19343058 PMC4002277

[B43] Echeverry C. Arredondo F. Abin-Carriquiry J. A. Midiwo J. O. Ochieng C. Kerubo L. (2010). Pretreatment with Natural Flavones and Neuronal Cell Survival after Oxidative Stress: A Structure−Activity Relationship Study. J. Agric. Food Chem. 58 (4), 2111–2115. 10.1021/jf902951v 20095615

[B44] Elumalai P. Lakshmi S. (2016). Role of Quercetin Benefits in Neurodegeneration. Adv. Neurobiol. 12, 229–245. 10.1007/978-3-319-28383-8_12 27651256

[B45] Esposito Z. Belli L. Toniolo S. Sancesario G. Bianconi C. Martorana A. (2013). Amyloid β, Glutamate, Excitotoxicity in Alzheimer's Disease: Are We on the Right Track? CNS Neurosci. Ther. 19 (8), 549–555. 10.1111/cns.12095 23593992 PMC6493397

[B46] FDA (2010). Agency Response Letter GRAS Notice No. GRN 000341. White Oak, MD: U.S. Food and Drug Administration (FDA).

[B47] Feng Y. Wang X. (2012). Antioxidant Therapies for Alzheimer's Disease. Oxidative Med. Cell Longevity 2012, 1–17. 10.1155/2012/472932 PMC3410354 22888398

[B48] Fischer C. Speth V. Fleig-Eberenz S. Neuhaus G. (1997). Induction of Zygotic Polyembryos in Wheat: Influence of Auxin Polar Transport. The Plant cell 9 (10), 1767–1780. 10.1105/tpc.9.10.176710.2307/3870523 12237347 PMC157020

[B49] Gandhi S. Abramov A. Y. (2012). Mechanism of Oxidative Stress in Neurodegeneration. Oxidative Med. Cell Longevity 2012, 1–11. 10.1155/2012/428010 PMC3362933 22685618

[B50] Gilgun-Sherki Y. Melamed E. Offen D. (2001). Oxidative Stress Induced-Neurodegenerative Diseases: the Need for Antioxidants that Penetrate the Blood Brain Barrier. Neuropharmacology 40 (8), 959–975. 10.1016/s0028-3908(01)00019-3 11406187

[B51] Glass C. K. Saijo K. Winner B. Marchetto M. C. Gage F. H. (2010). Mechanisms Underlying Inflammation in Neurodegeneration. Cell 140 (6), 918–934. 10.1016/j.cell.2010.02.016 20303880 PMC2873093

[B52] Gräff J. Kahn M. Samiei A. Gao J. Ota K. T. Rei D. (2013). A Dietary Regimen of Caloric Restriction or Pharmacological Activation of SIRT1 to Delay the Onset of Neurodegeneration. J. Neurosci. 33 (21), 8951–8960. 10.1523/jneurosci.5657-12.2013 23699506 PMC3775567

[B53] Graham D. I. Raghupathi R. Saatman K. E. Meaney D. McIntosh T. K. (2000). Tissue Tears in the white Matter after Lateral Fluid Percussion Brain Injury in the Rat: Relevance to Human Brain Injury. Acta Neuropathol. 99 (2), 117–124. 10.1007/pl00007414 10672317

[B54] Granado-Serrano A. B. Martín M. A. Bravo L. Goya L. Ramos S. (2010). Quercetin Modulates NF-κ B and AP-1/JNK Pathways to Induce Cell Death in Human Hepatoma Cells. Nutr. Cancer 62 (3), 390–401. 10.1080/01635580903441196 20358477

[B55] Greig N. H. Lahiri D. K. Sambamurti K. (2002). Butyrylcholinesterase: an Important New Target in Alzheimer's Disease Therapy. Int. Psychogeriatr. 14 (Suppl. 1), 77–91. 10.1017/s1041610203008676 12636181

[B56] Guo Y. Bruno R. S. (2015). Endogenous and Exogenous Mediators of Quercetin Bioavailability. J. Nutr. Biochem. 26 (3), 201–210. 10.1016/j.jnutbio.2014.10.008 25468612

[B57] Hanganu D. Olah N. K. Pop C. E. Vlase L. Oniga I. Ciocarlan N. (2019). EVALUATION OF POLYPHENOLIC PROFILE AND ANTIOXIDANT ACTIVITY FOR SOME SALVIA SPECIES. Farmacia 67 (5), 801–805. 10.31925/farmacia.2019.5.8

[B58] Hayakawa M. Itoh M. Ohta K. Li S. Ueda M. Wang M.-x. (2015). Quercetin Reduces eIF2α Phosphorylation by GADD34 Induction. Neurobiol. Aging 36 (9), 2509–2518. 10.1016/j.neurobiolaging.2015.05.006 26070242

[B59] Hendriks J. J. A. de Vries H. E. van der Pol S. M. A. van den Berg T. K. van Tol E. A. F. Dijkstra C. D. (2003). Flavonoids Inhibit Myelin Phagocytosis by Macrophages; a Structure-Activity Relationship Study. Biochem. Pharmacol. 65 (5), 877–885. 10.1016/s0006-2952(02)01609-x 12628496

[B60] Heneka M. T. Carson M. J. Khoury J. E. Landreth G. E. Brosseron F. Feinstein D. L. (2015). Neuroinflammation in Alzheimer's Disease. Lancet Neurol. 14 (4), 388–405. 10.1016/s1474-4422(15)70016-5 25792098 PMC5909703

[B61] Hickman S. E. Kingery N. D. Ohsumi T. K. Borowsky M. L. Wang L.-c. Means T. K. (2013). The Microglial Sensome Revealed by Direct RNA Sequencing. Nat. Neurosci. 16 (12), 1896–1905. 10.1038/nn.3554 24162652 PMC3840123

[B62] Ho L. Ferruzzi M. G. Janle E. M. Wang J. Gong B. Chen T. Y. (2013). Identification of Brain‐targeted Bioactive Dietary Quercetin‐3‐ O ‐glucuronide as a Novel Intervention for Alzheimer's Disease. FASEB j. 27 (2), 769–781. 10.1096/fj.12-212118 23097297 PMC3545533

[B63] Holzmann I. da Silva L. M. Corrêa da Silva J. A. Steimbach V. M. B. de Souza M. M. (2015). Antidepressant-like Effect of Quercetin in Bulbectomized Mice and Involvement of the Antioxidant Defenses, and the Glutamatergic and Oxidonitrergic Pathways. Pharmacol. Biochem. Behav. 136, 55–63. 10.1016/j.pbb.2015.07.003 26196245

[B64] Hooten K. G. Beers D. R. Zhao W. Appel S. H. (2015). Protective and Toxic Neuroinflammation in Amyotrophic Lateral Sclerosis. Neurotherapeutics 12 (2), 364–375. 10.1007/s13311-014-0329-3 25567201 PMC4404435

[B65] Hou Y. Aboukhatwa M. A. Lei D.-L. Manaye K. Khan I. Luo Y. (2010). Anti-depressant Natural Flavonols Modulate BDNF and Beta Amyloid in Neurons and hippocampus of Double TgAD Mice. Neuropharmacology 58 (6), 911–920. 10.1016/j.neuropharm.2009.11.002 19917299 PMC2838959

[B66] Hsiao H.-M. Sapinoro R. E. Thatcher T. H. Croasdell A. Levy E. P. Fulton R. A. (2013). A Novel Anti-inflammatory and Pro-resolving Role for Resolvin D1 in Acute Cigarette Smoke-Induced Lung Inflammation. PloS one 8 (3), e58258. 10.1371/journal.pone.0058258 23484005 PMC3590122

[B67] Inestrosa N. C. Alvarez A. Godoy J. Reyes A. De Ferrari G. V. (2000). Acetylcholinesterase-amyloid-β-peptide Interaction and Wnt Signaling Involvement in Aβ Neurotoxicity. Acta Neurol. Scand. Suppl. 102, 53–59. 10.1034/j.1600-0404.2000.00308.x 11261806

[B68] Ishige K. Schubert D. Sagara Y. (2001). Flavonoids Protect Neuronal Cells from Oxidative Stress by Three Distinct Mechanisms. Free Radic. Biol. Med. 30 (4), 433–446. 10.1016/s0891-5849(00)00498-6 11182299

[B69] Islam M. R. Zaman A. Jahan I. Chakravorty R. Chakraborty S. (2013). In Silico QSAR Analysis of Quercetin Reveals its Potential as Therapeutic Drug for Alzheimer's Disease. J. Young Pharm. 5 (4), 173–179. 10.1016/j.jyp.2013.11.005 24563598 PMC3930111

[B70] Jain D. Gangshettiwar A. (2014). Combination of Lycopene, Quercetin and Poloxamer188 Alleviates Anxiety and Depression in 3-nitropropionic Acid-Induced Huntingtons Disease in Rats. J. Intercult Ethnopharmacol 3 (4), 186–191. 10.5455/jice.20140903012921 26401371 PMC4576812

[B71] Jiang L. Kundu S. Lederman J. D. López-Hernández G. Y. Ballinger E. C. Wang S. (2016). Cholinergic Signaling Controls Conditioned Fear Behaviors and Enhances Plasticity of Cortical-Amygdala Circuits. Neuron 90 (5), 1057–1070. 10.1016/j.neuron.2016.04.028 27161525 PMC4891303

[B72] Jiménez-Aliaga K. Bermejo-Bescós P. Benedí J. Martín-Aragón S. (2011). Quercetin and Rutin Exhibit Antiamyloidogenic and Fibril-Disaggregating Effects *In Vitro* and Potent Antioxidant Activity in APPswe Cells. Life Sci. 89 (25-26), 939–945. 10.1016/j.lfs.2011.09.023 22008478

[B73] Johnson J. L. Rupasinghe S. G. Stefani F. Schuler M. A. Gonzalez de Mejia E. (2011). Citrus Flavonoids Luteolin, Apigenin, and Quercetin Inhibit Glycogen Synthase Kinase-3β Enzymatic Activity by Lowering the Interaction Energy within the Binding Cavity. J. Med. Food 14 (4), 325–333. 10.1089/jmf.2010.0310 21443429 PMC3123937

[B74] Jung M. Park M. (2007). Acetylcholinesterase Inhibition by Flavonoids from Agrimonia Pilosa. Molecules 12 (9), 2130–2139. 10.3390/12092130 17962731 PMC6149129

[B75] Jung S.-Y. Kim H.-J. Lee J.-Y. Cho J.-S. Lee Y.-S. Jin C.-B. (2012). Neuroprotective Effects of Quercetin 3-O-Methyl Ether, Quercetin and (±)-Dihydroquercetin in a Rat Model of Transient Focal Cerebral Ischemia. Bull. Korean Chem. Soc. 33, 2443–2446. 10.5012/bkcs.2012.33.7.2443

[B76] Jung Y.-H. Heo J. Lee Y. J. Kwon T. K. Kim Y.-H. (2010). Quercetin Enhances TRAIL-Induced Apoptosis in Prostate Cancer Cells via Increased Protein Stability of Death Receptor 5. Life Sci. 86 (9-10), 351–357. 10.1016/j.lfs.2010.01.008 20096292 PMC3003259

[B77] Kaariainen T. M. Piltonen M. Ossola B. Kekki H. Lehtonen Š. Nenonen T. (2008). Lack of Robust Protective Effect of Quercetin in Two Types of 6-Hydroxydopamine-Induced Parkinsonian Models in Rats and Dopaminergic Cell Cultures. Brain Res. 1203, 149–159. 10.1016/j.brainres.2008.01.089 18329008

[B78] Kandere-Grzybowska K. Kempuraj D. Cao J. Cetrulo C. L. Theoharides T. C. (2006). Regulation of IL-1-induced Selective IL-6 Release from Human Mast Cells and Inhibition by Quercetin. Br. J. Pharmacol. 148 (2), 208–215. 10.1038/sj.bjp.0706695 16532021 PMC1617055

[B79] Karuppagounder S. S. Madathil S. K. Pandey M. Haobam R. Rajamma U. Mohanakumar K. P. (2013). Quercetin Up-Regulates Mitochondrial Complex-I Activity to Protect against Programmed Cell Death in Rotenone Model of Parkinson's Disease in Rats. Neuroscience 236, 136–148. 10.1016/j.neuroscience.2013.01.032 23357119

[B80] Kawabata K. Mukai R. Ishisaka A. (2015). Quercetin and Related Polyphenols: New Insights and Implications for Their Bioactivity and Bioavailability. Food Funct. 6 (5), 1399–1417. 10.1039/c4fo01178c 25761771

[B81] Khan H. Ullah H. Aschner M. Cheang W. S. Akkol E. K. (2020). Neuroprotective Effects of Quercetin in Alzheimer's Disease. Biomolecules 10 (1), 59. 10.3390/biom10010059 PMC7023116 31905923

[B82] Kim H. Park B.-S. Lee K.-G. Choi C. Y. Jang S. S. Kim Y.-H. (2005). Effects of Naturally Occurring Compounds on Fibril Formation and Oxidative Stress of β-Amyloid. J. Agric. Food Chem. 53 (22), 8537–8541. 10.1021/jf051985c 16248550

[B83] Kim Y. J. Park W. (2016). Anti‐Inflammatory Effect of Quercetin on RAW 264.7 Mouse Macrophages Induced with Polyinosinic‐Polycytidylic Acid. Molecules 21 (4), 450. 10.3390/molecules21040450 27049378 PMC6273652

[B84] Kothari D. Lee W.-D. Kim S.-K. (2020). Allium Flavonols: Health Benefits, Molecular Targets, and Bioavailability. Antioxidants 9 (9), 888. 10.3390/antiox9090888 PMC7555649 32961762

[B85] Kuhad A. Singla S. Arora V. Chopra K. (2013). Neuroprotective Effect of Sesamol and Quercetin against QA Induced Neurotoxicity: An Experimental Paradigm of Huntington's Disease. J. Neurol. Sci. 333, e149–e150. 10.1016/j.jns.2013.07.498

[B86] Kumar A. Sehgal N. Kumar P. Padi S. S. V. Naidu P. S. (2008). Protective Effect of Quercetin against ICV Colchicine-Induced Cognitive Dysfunctions and Oxidative Damage in Rats. Phytother. Res. 22 (12), 1563–1569. 10.1002/ptr.2454 18980205

[B87] Lee S.-T. Chu K. Jung K.-H. Kim S.-J. Kim D.-H. Kang K.-M. (2008). Anti-inflammatory Mechanism of Intravascular Neural Stem Cell Transplantation in Haemorrhagic Stroke. Brain 131 (Pt 3), 616–629. 10.1093/brain/awm306 18156155

[B88] Lee Y. H. Kim H. J. Yoo H. Jung S. Y. Kwon B. J. Kim N.-J. (2015). Synthesis of (2-amino)ethyl Derivatives of Quercetin 3-O-Methyl Ether and Their Antioxidant and Neuroprotective Effects. Bioorg. Med. Chem. 23 (15), 4970–4979. 10.1016/j.bmc.2015.05.023 26068017

[B89] Lei X. Chao H. Zhang Z. Lv J. Li S. Wei H. (2015). Neuroprotective Effects of Quercetin in a Mouse Model of Brain Ischemic/reperfusion Injury via Anti-apoptotic Mechanisms Based on the Akt Pathway. Mol. Med. Rep. 12 (3), 3688–3696. 10.3892/mmr.2015.3857 26016839

[B90] Liu J. Yu H. Ning X. (2006). Effect of Quercetin on Chronic Enhancement of Spatial Learning and Memory of Mice. Sci. China Ser. C 49 (6), 583–590. 10.1007/s11427-006-2037-7 17312997

[B91] Liu Z. Tao X. Zhang C. Lu Y. Wei D. (2005). Protective Effects of Hyperoside (Quercetin-3-o-galactoside) to PC12 Cells against Cytotoxicity Induced by Hydrogen Peroxide and Tert-Butyl Hydroperoxide. Biomed. Pharmacother. 59 (9), 481–490. 10.1016/j.biopha.2005.06.009 16271843

[B92] Lu J. Wu D.-m. Zheng Y.-l. Hu B. Zhang Z.-f. Shan Q. (2010). Quercetin Activates AMP-Activated Protein Kinase by Reducing PP2C Expression Protecting Old Mouse Brain against High Cholesterol-Induced Neurotoxicity. J. Pathol. 222 (2), 199–212. 10.1002/path.2754 20690163

[B93] Lu J. Zheng Y. Luo L. Wu D. Sun D. Feng Y. (2006). Quercetin Reverses D-Galactose Induced Neurotoxicity in Mouse Brain. Behav. Brain Res. 171 (2), 251–260. 10.1016/j.bbr.2006.03.043 16707173

[B94] Lv C. Hong T. Yang Z. Zhang Y. Wang L. Dong M. (2012). Effect of Quercetin in the 1-Methyl-4-Phenyl-1, 2, 3, 6-Tetrahydropyridine-Induced Mouse Model of Parkinson's Disease. Evidence-Based Complement. Altern. Med. 2012, 1–6. 10.1155/2012/928643 PMC3290831 22454690

[B95] Lyman M. Lloyd D. G. Ji X. Vizcaychipi M. P. Ma D. (2014). Neuroinflammation: the Role and Consequences. Neurosci. Res. 79, 1–12. 10.1016/j.neures.2013.10.004 24144733

[B96] Madhavan P. N. N. Zainulabedin M. S. Nimisha H. G. Ramchand C. N. (2009). The Flavonoid, Quercetin, Inhibits HIV-1 Infection in Normal Peripheral Blood Mononuclear Cells. Am. J. Infect. Dis. 5 (2), 135–141. 10.3844/ajidsp.2009.135.141

[B97] Magalingam K. B. Radhakrishnan A. Haleagrahara N. (2016). Protective Effects of Quercetin Glycosides, Rutin, and Isoquercetrin against 6-hydroxydopamine (6-Ohda)-Induced Neurotoxicity in Rat Pheochromocytoma (PC-12) Cells. Int. J. Immunopathol Pharmacol. 29 (1), 30–39. 10.1177/0394632015613039 26542606 PMC5806739

[B98] Magalingam K. B. Radhakrishnan A. Ping N. S. Haleagrahara N. (2018). Current Concepts of Neurodegenerative Mechanisms in Alzheimer's Disease. Biomed. Res. Int. 2018, 1–12. 10.1155/2018/3740461 PMC5863339 29707568

[B99] Magalingam K. Radhakrishnan A. Haleagrahara N. (2014). Protective Effects of Flavonol Isoquercitrin, against 6-hydroxy Dopamine (6-OHDA) - Induced Toxicity in PC12 Cells. BMC Res. Notes 7, 49. 10.1186/1756-0500-7-49 24443837 PMC3910241

[B100] Marshall F. (2001). Heterodimerization of G-Protein-Coupled Receptors in the CNS. Curr. Opin. Pharmacol. 1 (1), 40–44. 10.1016/s1471-4892(01)00001-7 11712533

[B101] Mehdizadeh M. Taghi Joghataei M. Nobakht M. Aryanpour R. (2009). Neuroprotective Effect of Quercetin in a Model of Parkinson’s Disease in Rat: A Histochemical Analysis. BCN 1 (1), 3–6.

[B102] Mohammadi H. S. Goudarzi I. Lashkarbolouki T. Abrari K. Elahdadi Salmani M. (2014). Chronic Administration of Quercetin Prevent Spatial Learning and Memory Deficits Provoked by Chronic Stress in Rats. Behav. Brain Res. 270, 196–205. 10.1016/j.bbr.2014.05.015 24844750

[B103] Moore K. MacSween M. Shoichet M. (2006). Immobilized Concentration Gradients of Neurotrophic Factors Guide Neurite Outgrowth of Primary Neurons in Macroporous Scaffolds. Tissue Eng. 12 (2), 267–278. 10.1089/ten.2006.12.267 16548685

[B104] Moosavi F. Hosseini R. Saso L. Firuzi O. (2016). Modulation of Neurotrophic Signaling Pathways by Polyphenols. Drug Des. Devel Ther. 10, 23–42. 10.2147/dddt.S96936 PMC4694682 26730179

[B105] Mrvová N. Škandík M. Kuniaková M. Račková L. (2015). Modulation of BV-2 Microglia Functions by Novel Quercetin Pivaloyl Ester. Neurochem. Int. 90, 246–254. 10.1016/j.neuint.2015.09.005 26386394

[B106] Murray A. Faraoni M. Castro M. Alza N. Cavallaro V. (2013). Natural AChE Inhibitors from Plants and Their Contribution to Alzheimer's Disease Therapy. Cn 11 (4), 388–413. 10.2174/1570159X11311040004 PMC3744903 24381530

[B107] Naegele M. Martin R. (2014). The Good and the Bad of Neuroinflammation in Multiple Sclerosis. Handb Clin. Neurol. 122, 59–87. 10.1016/b978-0-444-52001-2.00003-0 24507513

[B108] Nakajima K.-i. Niisato N. Marunaka Y. (2011). Quercetin Stimulates NGF-Induced Neurite Outgrowth in PC12 Cells via Activation of Na+/K+/2Cl- Cotransporter. Cell Physiol Biochem 28 (1), 147–156. 10.1159/000331723 21865857

[B109] Naseri N. Valizadeh H. Zakeri-Milani P. (2015). Solid Lipid Nanoparticles and Nanostructured Lipid Carriers: Structure, Preparation and Application. Adv. Pharm. Bull. 5 (3), 305–313. 10.15171/apb.2015.043 26504751 PMC4616893

[B110] Nassiri-Asl M. Hajiali F. Taghiloo M. Abbasi E. Mohseni F. Yousefi F. (2016). Comparison between the Effects of Quercetin on Seizure Threshold in Acute and Chronic Seizure Models. Toxicol. Ind. Health 32 (5), 936–944. 10.1177/0748233713518603 24442347

[B111] Nassiri-Asl M. Moghbelinejad S. Abbasi E. Yonesi F. Haghighi M.-R. Lotfizadeh M. (2013). Effects of Quercetin on Oxidative Stress and Memory Retrieval in Kindled Rats. Epilepsy Behav. 28 (2), 151–155. 10.1016/j.yebeh.2013.04.019 23747498

[B112] Nazir A. Jadiya P. (2013). Sirtuin Mediated Neuroprotection and its Association with Autophagy and Apoptosis: Studies Employing Transgenic *C. elegans* Model. Mol. Neurodegeneration 8 (Suppl. 1), P65. 10.1186/1750-1326-8-s1-p65

[B113] Nichols M. Zhang J. Polster B. M. Elustondo P. A. Thirumaran A. Pavlov E. V. (2015). Synergistic Neuroprotection by Epicatechin and Quercetin: Activation of Convergent Mitochondrial Signaling Pathways. Neuroscience 308, 75–94. 10.1016/j.neuroscience.2015.09.012 26363153

[B114] Nieoczym D. Socała K. Raszewski G. Wlaź P. (2014). Effect of Quercetin and Rutin in Some Acute Seizure Models in Mice. Prog. Neuro-Psychopharmacology Biol. Psychiatry 54, 50–58. 10.1016/j.pnpbp.2014.05.007 24857758

[B115] Nieoullon A. (2011). Neurodegenerative Diseases and Neuroprotection: Current Views and Prospects. Jab 9 (4), 173–183. 10.2478/v10136-011-0013-4

[B116] Niranjan R. (2014). The Role of Inflammatory and Oxidative Stress Mechanisms in the Pathogenesis of Parkinson's Disease: Focus on Astrocytes. Mol. Neurobiol. 49 (1), 28–38. 10.1007/s12035-013-8483-x 23783559

[B117] Nixon R. A. (2013). The Role of Autophagy in Neurodegenerative Disease. Nat. Med. 19 (8), 983–997. 10.1038/nm.3232 23921753

[B118] Nussbaum L. Hogea L. Calina D. Andreescu N. Gradinaru R. Ștefănescu R. (2017). Modern Treatment Approaches in Psychoses. Pharmacogenetic, Neuroimagistic and Clinical Implications. Farmacia 65, 75–81.

[B119] Oliveira A. I. Pinho C. Sarmento B. Dias A. C. P. (2021). Quercetin-biapigenin Nanoparticles Are Effective to Penetrate the Blood-Brain Barrier. Drug Deliv. Transl. Res. 10.1007/s13346-021-00917-6 33709285

[B120] Omi N. Shiba H. Nishimura E. Tsukamoto S. Maruki-Uchida H. Oda M. (2019). Effects of Enzymatically Modified Isoquercitrin in Supplementary Protein Powder on Athlete Body Composition: a Randomized, Placebo-Controlled, Double-Blind Trial. J. Int. Soc. Sports Nutr. 16 (1), 39. 10.1186/s12970-019-0303-x 31500646 PMC6734270

[B121] Ono K. Yoshiike Y. Takashima A. Hasegawa K. Naiki H. Yamada M. (2003). Potent Anti-amyloidogenic and Fibril-Destabilizing Effects of Polyphenols *In Vitro*: Implications for the Prevention and Therapeutics of Alzheimer's Disease. J. Neurochem. 87 (1), 172–181. 10.1046/j.1471-4159.2003.01976.x 12969264

[B122] Padureanu R. Albu C. V. Mititelu R. R. Bacanoiu M. V. Docea A. O. Calina D. (2019). Oxidative Stress and Inflammation Interdependence in Multiple Sclerosis. Jcm 8 (11), 1815. 10.3390/jcm8111815 PMC6912446 31683787

[B123] Palazzolo G. Horvath P. Zenobi-Wong M. (2012). The Flavonoid Isoquercitrin Promotes Neurite Elongation by Reducing RhoA Activity. PLoS One 7 (11), e49979. 10.1371/journal.pone.0049979 23209630 PMC3510166

[B124] Pany S. Pal A. Sahu P. K. (2014). Neuroprotective Effect of Quercetin in Neurotoxicity Induced Rats: Role of Neuroinflammation in Neurodegeneration. Asian J. Pharm. Clin. Res. 7 (4), 152–156.

[B125] Parsons C. G. Danysz W. Dekundy A. Pulte I. (2013). Memantine and Cholinesterase Inhibitors: Complementary Mechanisms in the Treatment of Alzheimer's Disease. Neurotox Res. 24 (3), 358–369. 10.1007/s12640-013-9398-z 23657927 PMC3753463

[B126] Pateiro M. Gómez B. Munekata P. E. S. Barba F. J. Putnik P. Kovačević D. B. (2021). Nanoencapsulation of Promising Bioactive Compounds to Improve Their Absorption, Stability, Functionality and the Appearance of the Final Food Products. Molecules 26 (6), 1547. 10.3390/molecules26061547 33799855 PMC7999092

[B127] Pinheiro R. G. R. Granja A. Loureiro J. A. Pereira M. C. Pinheiro M. Neves A. R. (2020). Quercetin Lipid Nanoparticles Functionalized with Transferrin for Alzheimer's Disease. Eur. J. Pharm. Sci. 148 **,** 105314. 10.1016/j.ejps.2020.105314 32200044

[B128] Portelius E. Zetterberg H. Andreasson U. Brinkmalm G. Andreasen N. Wallin A. (2006). An Alzheimer's Disease-specific β-amyloid Fragment Signature in Cerebrospinal Fluid. Neurosci. Lett. 409 (3), 215–219. 10.1016/j.neulet.2006.09.044 17049739

[B129] Prasad J. Baitharu I. Sharma A. K. Dutta R. Prasad D. Singh S. B. (2013). Quercetin Reverses Hypobaric Hypoxia-Induced Hippocampal Neurodegeneration and Improves Memory Function in the Rat. High Alt. Med. Biol. 14 (4), 383–394. 10.1089/ham.2013.1014 24377346

[B130] Qu L. Liang X. Gu B. Liu W. (2014). Quercetin Alleviates High Glucose-Induced Schwann Cell Damage by Autophagy. Neural Regen. Res. 9 (12), 1195–1203. 10.4103/1673-5374.135328 25206782 PMC4146282

[B131] Rasool M. Malik A. Qureshi M. S. Manan A. Pushparaj P. N. Asif M. (2014). Recent Updates in the Treatment of Neurodegenerative Disorders Using Natural Compounds. Evidence-Based Complement. Altern. Med. 2014, 1–7. 10.1155/2014/979730 PMC4017872 24864161

[B132] Regitz C. Marie Dußling L. Wenzel U. (2014). Amyloid-beta (Aβ1-42)-Induced Paralysis inCaenorhabditis Elegansis Inhibited by the Polyphenol Quercetin through Activation of Protein Degradation Pathways. Mol. Nutr. Food Res. 58 (10), 1931–1940. 10.1002/mnfr.201400014 25066301

[B133] Rego A. C. Oliveira C. R. (2003). Mitochondrial Dysfunction and Reactive Oxygen Species in Excitotoxicity and Apoptosis: Implications for the Pathogenesis of Neurodegenerative Diseases. Neurochem. Res. 28 (10), 1563–1574. 10.1023/a:1025682611389 14570402

[B134] Richetti S. K. Blank M. Capiotti K. M. Piato A. L. Bogo M. R. Vianna M. R. (2011). Quercetin and Rutin Prevent Scopolamine-Induced Memory Impairment in Zebrafish. Behav. Brain Res. 217 (1), 10–15. 10.1016/j.bbr.2010.09.027 20888863

[B135] Riva A. Ronchi M. Petrangolini G. Bosisio S. Allegrini P. (2019). Improved Oral Absorption of Quercetin from Quercetin Phytosome, a New Delivery System Based on Food Grade Lecithin. Eur. J. Drug Metab. Pharmacokinet. 44 (2), 169–177. 10.1007/s13318-018-0517-3 30328058 PMC6418071

[B136] Rocha N. P. de Miranda A. S. Teixeira A. L. (2015). Insights into Neuroinflammation in Parkinson's Disease: From Biomarkers to Anti-inflammatory Based Therapies. Biomed. Res. Int. 2015, 1–12. 10.1155/2015/628192 PMC4532803 26295044

[B137] Sabogal-Guáqueta A. M. Muñoz-Manco J. I. Ramírez-Pineda J. R. Lamprea-Rodriguez M. Osorio E. Cardona-Gómez G. P. (2015). The Flavonoid Quercetin Ameliorates Alzheimer's Disease Pathology and Protects Cognitive and Emotional Function in Aged Triple Transgenic Alzheimer's Disease Model Mice. Neuropharmacology 93, 134–145. 10.1016/j.neuropharm.2015.01.027 25666032 PMC4387064

[B138] Sabri O. Kendziorra K. Wolf H. Gertz H.-J. Brust P. (2008). Acetylcholine Receptors in Dementia and Mild Cognitive Impairment. Eur. J. Nucl. Med. Mol. Imaging 35 (Suppl. 1), 30–45. 10.1007/s00259-007-0701-1 18228017

[B139] Saha K. Lajis N. H. Israf D. A. Hamzah A. S. Khozirah S. Khamis S. (2004). Evaluation of Antioxidant and Nitric Oxide Inhibitory Activities of Selected Malaysian Medicinal Plants. J. Ethnopharmacology 92 (2-3), 263–267. 10.1016/j.jep.2004.03.007 15138010

[B140] Salazar C. Höfer T. (2009). Multisite Protein Phosphorylation - from Molecular Mechanisms to Kinetic Models. Febs j 276 (12), 3177–3198. 10.1111/j.1742-4658.2009.07027.x 19438722

[B141] Salehi B. Calina D. Docea A. Koirala N. Aryal S. Lombardo D. (2020a). Curcumin's Nanomedicine Formulations for Therapeutic Application in Neurological Diseases. Jcm 9 (2), 430. 10.3390/jcm9020430 PMC7074182 32033365

[B142] Salehi B. Capanoglu E. Adrar N. Catalkaya G. Shaheen S. Jaffer M. (2019a). Cucurbits Plants: A Key Emphasis to its Pharmacological Potential. Molecules 24 (10), 1854. 10.3390/molecules24101854 PMC6572650 31091784

[B143] Salehi B. Prakash Mishra A. Nigam M. Karazhan N. Shukla I. Kiełtyka‐Dadasiewicz A. (2021a). Ficus Plants: State of the Art from a Phytochemical, Pharmacological, and Toxicological Perspective. Phytotherapy Res. 35, 1187–1217. 10.1002/ptr.6884 33025667

[B144] Salehi B. Quispe C. Chamkhi I. El Omari N. Balahbib A. Sharifi-Rad J. (2021b). Pharmacological Properties of Chalcones: A Review of Preclinical Including Molecular Mechanisms and Clinical Evidence. Front. Pharmacol. 11, 592654. 10.3389/fphar.2020.592654 33536909 PMC7849684

[B145] Salehi B. Rescigno A. Dettori T. Calina D. Docea A. O. Singh L. (2020b). Avocado-Soybean Unsaponifiables: A Panoply of Potentialities to Be Exploited. Biomolecules 10 (1), 130. 10.3390/biom10010130 PMC7023362 31940989

[B146] Salehi B. Sestito S. Rapposelli S. Peron G. Calina D. Sharifi-Rad M. (2019b). Epibatidine: A Promising Natural Alkaloid in Health. Biomolecules 9 (1), 6. 10.3390/biom9010006 PMC6359223 30583611

[B147] Salehi B. Sharifi-Rad J. Cappellini F. Reiner Ž. Zorzan D. Imran M. (2020c). The Therapeutic Potential of Anthocyanins: Current Approaches Based on Their Molecular Mechanism of Action. Front. Pharmacol. 11, 1300. 10.3389/fphar.2020.01300 32982731 PMC7479177

[B148] Salehi B. Shivaprasad Shetty M. V. Anil Kumar N. Živković J. Calina D. Oana Docea A. (2019c). Veronica Plants-Drifting from Farm to Traditional Healing, Food Application, and Phytopharmacology. Molecules 24 (13), 2454. 10.3390/molecules24132454 PMC6651156 31277407

[B149] Salloway S. Mintzer J. Weiner M. F. Cummings J. L. (2008). Disease-modifying Therapies in Alzheimer's Disease. Alzheimer's Demen. 4 (2), ALZJJALZ200710001–79. 10.1016/j.jalz.2007.10.001 18631951

[B150] Sandhir R. Mehrotra A. (2013). Quercetin Supplementation Is Effective in Improving Mitochondrial Dysfunctions Induced by 3-nitropropionic Acid: Implications in Huntington's Disease. Biochim. Biophys. Acta (Bba) - Mol. Basis Dis. 1832 (3), 421–430. 10.1016/j.bbadis.2012.11.018 23220257

[B151] Sarkar A. Angeline M. S. Anand K. Ambasta R. K. Kumar P. (2012). Naringenin and Quercetin Reverse the Effect of Hypobaric Hypoxia and Elicit Neuroprotective Response in the Murine Model. Brain Res. 1481, 59–70. 10.1016/j.brainres.2012.08.036 22981402

[B152] Saul N. Pietsch K. Menzel R. Steinberg C. E. W. (2008). Quercetin-mediated Longevity in *Caenorhabditis elegans*: Is DAF-16 Involved? Mech. Ageing Development 129 (10), 611–613. 10.1016/j.mad.2008.07.001 18692520

[B153] Schültke E. Kamencic H. Skihar V. M. Griebel R. Juurlink B. (2010). Quercetin in an Animal Model of Spinal Cord Compression Injury: Correlation of Treatment Duration with Recovery of Motor Function. Spinal Cord 48 (2), 112–117. 10.1038/sc.2009.111 19736558

[B154] Sefil F. Kahraman I. Dokuyucu R. Gokce H. Ozturk A. Tutuk O. (2014). Ameliorating Effect of Quercetin on Acute Pentylenetetrazole Induced Seizures in Rats. Int. J. Clin. Exp. Med. 7 (9), 2471–2477. 25356099 PMC4211749

[B155] Selkoe D. J. (2001). Alzheimer's Disease Results from the Cerebral Accumulation and Cytotoxicity of Amyloid SS-Protein. Jad 3 (1), 75–80. 10.3233/jad-2001-3111 12214075

[B156] Sharifi-Rad J. Dey A. Koirala N. Shaheen S. El Omari N. Salehi B. (2021a). Cinnamomum Species: Bridging Phytochemistry Knowledge, Pharmacological Properties and Toxicological Safety for Health Benefits. Front. Pharmacol. 12, 600139. 10.3389/fphar.2021.600139 34045956 PMC8144503

[B157] Sharifi-Rad J. Kamiloglu S. Yeskaliyeva B. Beyatli A. Alfred M. A. Salehi B. (2020a). Pharmacological Activities of Psoralidin: A Comprehensive Review of the Molecular Mechanisms of Action. Front. Pharmacol. 11, 11. 10.3389/fphar.2020.571459 33192514 PMC7643726

[B158] Sharifi-Rad J. Quispe C. Shaheen S. El Haouari M. Azzini E. Butnariu M. (2021b). Flavonoids as Potential Anti-platelet Aggregation Agents: from Biochemistry to Health Promoting Abilities. Crit. Rev. Food Sci. Nutr., 1–14. 10.1080/10408398.2021.1924612 33983094

[B159] Sharifi-Rad J. Rodrigues C. F. Sharopov F. Docea A. O. Can Karaca A. Sharifi-Rad M. (2020b). Diet, Lifestyle and Cardiovascular Diseases: Linking Pathophysiology to Cardioprotective Effects of Natural Bioactive Compounds. Ijerph 17 (7), 2326. 10.3390/ijerph17072326 PMC7177934 32235611

[B160] Sharifi-Rad M. Anil Kumar N. V. Zucca P. Varoni E. M. Dini L. Panzarini E. (2020d). Lifestyle, Oxidative Stress, and Antioxidants: Back and Forth in the Pathophysiology of Chronic Diseases. Front. Physiol. 11, 694. 10.3389/fphys.2020.00694 32714204 PMC7347016

[B161] Sharifi-Rad M. Lankatillake C. Dias D. A. Docea A. O. Mahomoodally M. F. Lobine D. (2020e). Impact of Natural Compounds on Neurodegenerative Disorders: From Preclinical to Pharmacotherapeutics. Jcm 9 (4), 1061. 10.3390/jcm9041061 PMC7231062 32276438

[B162] Sharma D. R. Wani W. Y. Sunkaria A. Kandimalla R. J. L. Verma D. Cameotra S. S. (2013). Quercetin Protects against Chronic Aluminum-Induced Oxidative Stress and Ensuing Biochemical, Cholinergic, and Neurobehavioral Impairments in Rats. Neurotox Res. 23 (4), 336–357. 10.1007/s12640-012-9351-6 22918785

[B163] Sharma D. R. Wani W. Y. Sunkaria A. Kandimalla R. J. Sharma R. K. Verma D. (2016). Quercetin Attenuates Neuronal Death against Aluminum-Induced Neurodegeneration in the Rat hippocampus. Neuroscience 324, 163–176. 10.1016/j.neuroscience.2016.02.055 26944603

[B164] Sharma V. Mishra M. Ghosh S. Tewari R. Basu A. Seth P. (2007). Modulation of Interleukin-1β Mediated Inflammatory Response in Human Astrocytes by Flavonoids: Implications in Neuroprotection. Brain Res. Bull. 73 (1-3), 55–63. 10.1016/j.brainresbull.2007.01.016 17499637

[B165] Shi C. Zhao L. Zhu B. Li Q. Yew D. T. Yao Z. (2009). Protective Effects of Ginkgo Biloba Extract (EGb761) and its Constituents Quercetin and Ginkgolide B against β-amyloid Peptide-Induced Toxicity in SH-Sy5y Cells. Chemico-Biological Interactions 181 (1), 115–123. 10.1016/j.cbi.2009.05.010 19464278

[B166] Shigeri Y. Seal R. P. Shimamoto K. (2004). Molecular Pharmacology of Glutamate Transporters, EAATs and VGLUTs. Brain Res. Rev. 45 (3), 250–265. 10.1016/j.brainresrev.2004.04.004 15210307

[B167] Shimmyo Y. Kihara T. Akaike A. Niidome T. Sugimoto H. (2008). Flavonols and Flavones as BACE-1 Inhibitors: Structure-Activity Relationship in Cell-free, Cell-Based and In Silico Studies Reveal Novel Pharmacophore Features. Biochim. Biophys. Acta (Bba) - Gen. Subjects 1780 (5), 819–825. 10.1016/j.bbagen.2008.01.017 18295609

[B168] Shokoohinia Y. Rashidi M. Hosseinzadeh L. Jelodarian Z. (2015). Quercetin-3-O-β-d-glucopyranoside, a Dietary Flavonoid, Protects PC12 Cells from H2O2-Induced Cytotoxicity through Inhibition of Reactive Oxygen Species. Food Chem. 167, 162–167. 10.1016/j.foodchem.2014.06.079 25148973

[B169] Silva B. Oliveira P. J. Dias A. Malva J. O. (2008). Quercetin, Kaempferol and Biapigenin Fromhypericum Perforatum Are Neuroprotective against Excitotoxic Insults. Neurotox Res. 13 (3-4), 265–279. 10.1007/bf03033510 18522906

[B170] Singh A. Naidu P. S. Kulkarni S. K. (2003). Reversal of Aging and Chronic Ethanol-Induced Cognitive Dysfunction by Quercetin a Bioflavonoid. Free Radic. Res. 37 (11), 1245–1252. 10.1080/10715760310001616014 14703737

[B171] Siokas V. Aloizou A.-M. Tsouris Z. Liampas I. Liakos P. Calina D. (2021). ADORA2A Rs5760423 and CYP1A2 Rs762551 Polymorphisms as Risk Factors for Parkinson's Disease. Jcm 10 (3), 381. 10.3390/jcm10030381 33498513 PMC7864159

[B172] Smith J. A. Das A. Ray S. K. Banik N. L. (2012). Role of Pro-inflammatory Cytokines Released from Microglia in Neurodegenerative Diseases. Brain Res. Bull. 87 (1), 10–20. 10.1016/j.brainresbull.2011.10.004 22024597 PMC9827422

[B173] Smith J. V. Luo Y. (2003). Elevation of Oxidative Free Radicals in Alzheimer's Disease Models Can Be Attenuated by Ginkgo Biloba Extract EGb 761. Jad 5 (4), 287–300. 10.3233/jad-2003-5404 14624024

[B174] Sriraksa N. Wattanathorn J. Muchimapura S. Tiamkao S. Brown K. Chaisiwamongkol K. (2012). Cognitive-enhancing Effect of Quercetin in a Rat Model of Parkinson's Disease Induced by 6-hydroxydopamine. Evidence-Based Complement. Altern. Med. 2012, 1–9. 10.1155/2012/823206 PMC3139913 21792372

[B175] Sternberg Z. Chadha K. Lieberman A. Hojnacki D. Drake A. Zamboni P. (2008). Quercetin and Interferon-β Modulate Immune Response(s) in Peripheral Blood Mononuclear Cells Isolated from Multiple Sclerosis Patients. J. Neuroimmunology 205 (1-2), 142–147. 10.1016/j.jneuroim.2008.09.008 18926575

[B176] Suematsu N. Hosoda M. Fujimori K. (2011). Protective Effects of Quercetin against Hydrogen Peroxide-Induced Apoptosis in Human Neuronal SH-Sy5y Cells. Neurosci. Lett. 504 (3), 223–227. 10.1016/j.neulet.2011.09.028 21964380

[B177] Suganthy N. Devi K. P. Nabavi S. F. Braidy N. Nabavi S. M. (2016). Bioactive Effects of Quercetin in the central Nervous System: Focusing on the Mechanisms of Actions. Biomed. Pharmacother. 84 **,** 892–908. 10.1016/j.biopha.2016.10.011 27756054

[B178] Sun G. Y. Chen Z. Jasmer K. J. Chuang D. Y. Gu Z. Hannink M. (2015). Quercetin Attenuates Inflammatory Responses in BV-2 Microglial Cells: Role of MAPKs on the Nrf2 Pathway and Induction of Heme Oxygenase-1. PloS one 10 (10), e0141509. 10.1371/journal.pone.0141509 26505893 PMC4624710

[B179] Sun S. W. Yu H. Q. Zhang H. Zheng Y. L. Wang J. J. Luo L. (2007). Quercetin Attenuates Spontaneous Behavior and Spatial Memory Impairment in D-Galactose-Treated Mice by Increasing Brain Antioxidant Capacity. Nutr. Res. 27 (3), 169–175. 10.1016/j.nutres.2007.01.010

[B180] Szydlowska K. Tymianski M. (2010). Calcium, Ischemia and Excitotoxicity. Cell Calcium 47 (2), 122–129. 10.1016/j.ceca.2010.01.003 20167368

[B181] Tai H.-C. Serrano-Pozo A. Hashimoto T. Frosch M. P. Spires-Jones T. L. Hyman B. T. (2012). The Synaptic Accumulation of Hyperphosphorylated Tau Oligomers in Alzheimer Disease Is Associated with Dysfunction of the Ubiquitin-Proteasome System. Am. J. Pathol. 181 (4), 1426–1435. 10.1016/j.ajpath.2012.06.033 22867711 PMC3463637

[B182] Tangsaengvit N. Kitphati W. Tadtong S. Bunyapraphatsara N. Nukoolkarn V. (2013). Neurite Outgrowth and Neuroprotective Effects of Quercetin fromCaesalpinia mimosoidesLamk. On Cultured P19-Derived Neurons. Evidence-Based Complement. Altern. Med. 2013, 1–7. 10.1155/2013/838051 PMC3693115 23840266

[B183] Tay W. M. da Silva G. F. Z. Ming L.-J. (2013). Metal Binding of Flavonoids and Their Distinct Inhibition Mechanisms toward the Oxidation Activity of Cu2+-β-Amyloid: Not Just Serving as Suicide Antioxidants!. Inorg. Chem. 52 (2), 679–690. 10.1021/ic301832p 23301941

[B184] Tchantchou F. Lacor P. N. Cao Z. Lao L. Hou Y. Cui C. (2009). Stimulation of Neurogenesis and Synaptogenesis by Bilobalide and Quercetin via Common Final Pathway in Hippocampal Neurons. Jad 18 (4), 787–798. 10.3233/jad-2009-1189 19661619

[B185] Testa G. Gamba P. Badilli U. Gargiulo S. Maina M. Guina T. (2014). Loading into Nanoparticles Improves Quercetin's Efficacy in Preventing Neuroinflammation Induced by Oxysterols. PLoS One 9 (5), e96795. 10.1371/journal.pone.0096795 24802026 PMC4011877

[B186] Tsatsakis A. Docea A. O. Calina D. Tsarouhas K. Zamfira L. M. Mitrut R. (2019). A Mechanistic and Pathophysiological Approach for Stroke Associated with Drugs of Abuse. Jcm 8 (9), 1295. 10.3390/jcm8091295 PMC6780697 31450861

[B187] Tsoukalas D. Zlatian O. Mitroi M. Renieri E. Tsatsakis A. Izotov B. N. (2021). A Novel Nutraceutical Formulation Can Improve Motor Activity and Decrease the Stress Level in a Murine Model of Middle-Age Animals. Jcm 10 (4), 624. 10.3390/jcm10040624 33562115 PMC7915416

[B188] Uttara B. Singh A. Zamboni P. Mahajan R. (2009). Oxidative Stress and Neurodegenerative Diseases: a Review of Upstream and Downstream Antioxidant Therapeutic Options. Cn 7 (1), 65–74. 10.2174/157015909787602823 PMC2724665 19721819

[B189] Wang J.-Z. Liu F. (2008). Microtubule-associated Protein Tau in Development, Degeneration and protection of Neurons. Prog. Neurobiol. 85 (2), 148–175. 10.1016/j.pneurobio.2008.03.002 18448228

[B190] Wang S. Su R. Nie S. Sun M. Zhang J. Wu D. (2014). Application of Nanotechnology in Improving Bioavailability and Bioactivity of Diet-Derived Phytochemicals. J. Nutr. Biochem. 25 (4), 363–376. 10.1016/j.jnutbio.2013.10.002 24406273 PMC3959237

[B191] Waxman E. A. Giasson B. I. (2011). Characterization of Kinases Involved in the Phosphorylation of Aggregated α-synuclein. J. Neurosci. Res. 89 (2), 231–247. 10.1002/jnr.22537 21162130 PMC4484797

[B192] Waxman E. A. Giasson B. I. (2008). Specificity and Regulation of Casein Kinase-Mediated Phosphorylation of α-Synuclein. J. Neuropathol. Exp. Neurol. 67 (5), 402–416. 10.1097/NEN.0b013e31816fc99510.1097/nen.0b013e3186fc995 18451726 PMC2930078

[B193] Xia S.-F. Xie Z.-X. Qiao Y. Li L.-R. Cheng X.-R. Tang X. (2015). Differential Effects of Quercetin on Hippocampus-dependent Learning and Memory in Mice Fed with Different Diets Related with Oxidative Stress. Physiol. Behav. 138, 325–331. 10.1016/j.physbeh.2014.09.008 25447470

[B194] Yang D. Wang T. Long M. Li P. (2020). Quercetin: Its Main Pharmacological Activity and Potential Application in Clinical Medicine. Oxidative Med. Cell Longevity 2020, 1–13. 10.1155/2020/8825387 PMC7790550 33488935

[B195] Yang E. J. Kim G. S. Kim J. A. Song K. S. (2013). Protective Effects of Onion-Derived Quercetin on Glutamate-Mediated Hippocampal Neuronal Cell Death. Pharmacogn Mag. 9 (36), 302–308. 10.4103/0973-1296.117824 24124281 PMC3793334

[B196] Yao Y. Han D. D. Zhang T. Yang Z. (2010). Quercetin Improves Cognitive Deficits in Rats with Chronic Cerebral Ischemia and Inhibits Voltage-dependent Sodium Channels in Hippocampal CA1 Pyramidal Neurons. Phytother. Res. 24 (1), 136–140. 10.1002/ptr.2902 19688719

[B205] Yeung A. W. K. Tzvetkov N. T. Atanasov A. G. (2018). When Neuroscience Meets Pharmacology: A Neuropharmacology Literature Analysis. Front. Neurosci. 12, 852. 10.3389/fnins.2018.00852 30505266 PMC6250846

[B197] Youdim K. A. Qaiser M. Z. Begley D. J. Rice-Evans C. A. Abbott N. J. (2004). Flavonoid Permeability across an *In Situ* Model of the Blood-Brain Barrier. Free Radic. Biol. Med. 36 (5), 592–604. 10.1016/j.freeradbiomed.2003.11.023 14980703

[B198] Yu X. Li Y. Mu X. (2020). Effect of Quercetin on PC12 Alzheimer's Disease Cell Model Induced by Aβ25-35 and its Mechanism Based on Sirtuin1/Nrf2/HO-1 Pathway. Biomed. Res. Int. 2020, 1–10. 10.1155/2020/8210578 PMC7201675 32420373

[B199] Zaplatic E. Bule M. Shah S. Z. A. Uddin M. S. Niaz K. (2019). Molecular Mechanisms Underlying Protective Role of Quercetin in Attenuating Alzheimer's Disease. Life Sci. 224 **,** 109–119. 10.1016/j.lfs.2019.03.055 30914316

[B200] Zhang M. Swarts S. G. Yin L. Liu C. Tian Y. Cao Y. (2011). “Antioxidant Properties of Quercetin,” in Oxygen Transport to Tissue XXXII. Editors LaManna J. C. Puchowicz M. A. Xu K. Harrison D. K. (Springer US)), 283–289.()

[B201] Zhang X. D. Liu X. Q. Kim Y. H. Whang W. K. (2014). Chemical Constituents and Their Acetyl Cholinesterase Inhibitory and Antioxidant Activities from Leaves of Acanthopanax Henryi: Potential Complementary Source against Alzheimer's Disease. Arch. Pharm. Res. 37 (5), 606–616. 10.1007/s12272-013-0252-x 24085630

[B202] Zhang X. Hu J. Zhong L. Wang N. Yang L. Liu C.-C. (2016). Quercetin Stabilizes Apolipoprotein E and Reduces Brain Aβ Levels in Amyloid Model Mice. Neuropharmacology 108, 179–192. 10.1016/j.neuropharm.2016.04.032 27114256

[B203] Zheng J. Wu J. Chen J. Liu J. Lu Y. Huang C. (2016). Therapeutic Effects of Quercetin on Early Inflammation in Hypertriglyceridemia-Related Acute Pancreatitis and its Mechanism. Pancreatology 16 (2), 200–210. 10.1016/j.pan.2016.01.005 26873426

[B204] Zhu M. Han S. Fink A. L. (2013). Oxidized Quercetin Inhibits α-synuclein Fibrillization. Biochim. Biophys. Acta (Bba) - Gen. Subjects 1830 (4), 2872–2881. 10.1016/j.bbagen.2012.12.027 23295967

